# Computation-Aided Engineering of Cytochrome P450 for the Production of Pravastatin

**DOI:** 10.1021/acscatal.2c03974

**Published:** 2022-11-28

**Authors:** Mark A. Ashworth, Elvira Bombino, René M. de Jong, Hein J. Wijma, Dick B. Janssen, Kirsty J. McLean, Andrew W. Munro

**Affiliations:** † Manchester Institute of Biotechnology, School of Chemistry, The University of Manchester, Manchester M1 7DN, United Kingdom; ‡ Department of Biochemistry, Groningen Biomolecular Sciences and Biotechnology Institute, University of Groningen, Nijenborgh 4, Groningen 9747 AG, Netherlands; § DSM Food & Beverage, Alexander Fleminglaan 1, 2613 AX Delft, the Netherlands; ∥ Department of Biological and Geographical Sciences, School of Applied Sciences, University of Huddersfield, Huddersfield HD1 3DH, United Kingdom

**Keywords:** biocatalysis, chiral precursor, in silico screening, computational design, asymmetric synthesis, stereoselectivity, enzyme engineering

## Abstract

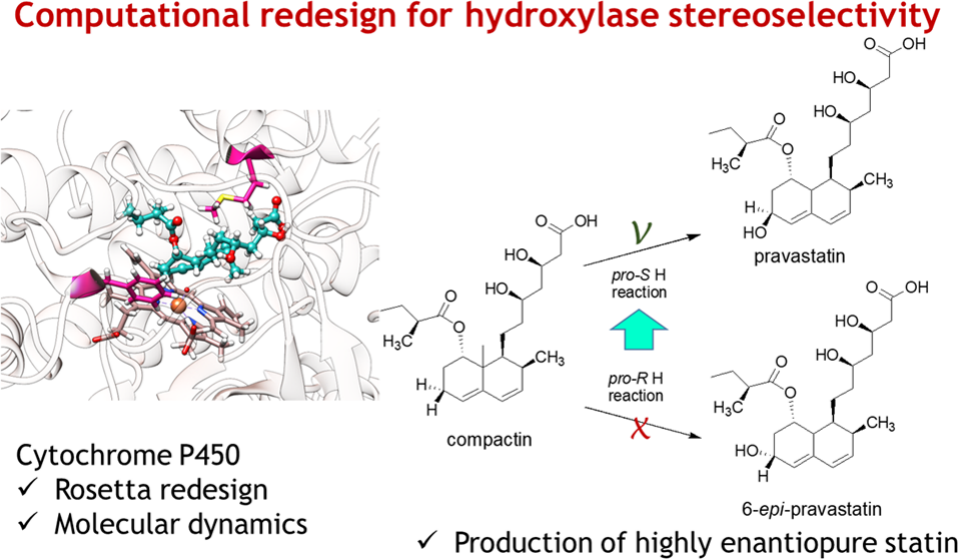

CYP105AS1 is a cytochrome P450 from *Amycolatopsis orientalis* that catalyzes monooxygenation of compactin to 6-epi-pravastatin. For fermentative production of the cholesterol-lowering drug pravastatin, the stereoselectivity of the enzyme needs to be inverted, which has been partially achieved by error-prone PCR mutagenesis and screening. In the current study, we report further optimization of the stereoselectivity by a computationally aided approach. Using the CoupledMoves protocol of Rosetta, a virtual library of mutants was designed to bind compactin in a pro-pravastatin orientation. By examining the frequency of occurrence of beneficial substitutions and rational inspection of their interactions, a small set of eight mutants was predicted to show the desired selectivity and these variants were tested experimentally. The best CYP105AS1 variant gave >99% stereoselective hydroxylation of compactin to pravastatin, with complete elimination of the unwanted 6-epi-pravastatin diastereomer. The enzyme–substrate complexes were also examined by ultrashort molecular dynamics simulations of 50 × 100 ps and 5 × 22 ns, which revealed that the frequency of occurrence of near-attack conformations agreed with the experimentally observed stereoselectivity. These results show that a combination of computational methods and rational inspection could improve CYP105AS1 stereoselectivity beyond what was obtained by directed evolution. Moreover, the work lays out a general *in silico* framework for specificity engineering of enzymes of known structure.

## Introduction

The use of enzymes as industrial catalysts is attractive to produce enantiomerically pure compounds through asymmetric synthesis or kinetic resolution of racemates. Enzymes catalyze reactions under mild conditions, which makes problems like isomerization or racemization of chiral compounds less likely. The therapeutic action of many chiral pharmaceutical compounds is dependent on the interaction of a single stereoisomer with a biological target.^1^ This includes statin drugs.^2^ Moreover, the presence of a noneffective stereoisomer can lead to unwanted side effects.^3^ Accordingly, methods for the synthesis of stereochemically pure formulations are required for the production of pharmaceutically active ingredients.^4^ Unfortunately, the applicability of many natural enzymes is hindered by low activity, modest specificity or stereoselectivity, or poor stability under process conditions. Molecular enzyme engineering techniques can overcome such limitations.^5^ Enzymes can be tailored to obtain a desired selectivity and to match process requirements, often through directed evolution.^6−8^ In this technique, mutant libraries are constructed by random or localized mutagenesis from which beneficial variants are selected by laboratory screening.^9^ However, screening of large numbers of variants for stereoselectivity remains a bottleneck in directed evolution campaigns. This makes it desirable to increase the occurrence of beneficial variants in libraries, *e.g.*, by using structural information.

Over the last decade, the use of computational approaches to design enzymes has received increasing attention. Better energy functions and search algorithms allow *de novo* design of protein structures, including active sites that recognize specific ligands and catalyze diverse reactions.^10−12^ Various enzyme design studies employ Rosetta energy scoring functions in combination with visual inspection of the designed structures to select the most promising variants.^10,13^ However, the design of highly active enzymes has met serious difficulties, often making it necessary to enhance the catalytic activity by directed evolution.^10,14^ On the other hand, computational approaches can also overcome some limitations of directed evolution, *e.g.*, by reducing the time and costs related to the number of enzyme variants that must be experimentally tested.^15−18^ A key challenge for *in silico* protocols is to develop widely applicable methodologies that can explore vast amounts of sequence space and yet have high prediction accuracy.^19−21^

The majority of predictive algorithms rely on fixed protein backbone approaches,^11,14,22−25^ with few exceptions where backbone movement is allowed, such as the enzyme design application in Rosetta.^26−29^ Further protein backbone movements were implemented by Ollikainen et al.^30^ The “backrub” algorithm in their CoupledMoves protocol allows inclusion of significant protein plasticity in Rosetta computational design.^30^ The algorithm couples changes in protein backbone conformations with side-chain rotamer substitutions before scoring their favorability. The CoupledMoves protocol thus can account for the importance of protein backbone conformations and protein flexibility when searching for low-energy designs.^30,31^ This can improve the accuracy of predicted protein–ligand complexes and was used to reproduce the results of enzyme engineering experiments and natural sequence diversity in enzyme families.^31^ However, the use of this protocol for the prediction and selection of new enzyme variants has not yet been reported.

Computational design protocols normally produce a large number of designs which should be ranked in subsequent steps to select the best candidates for experimental verification. To decrease the number of dysfunctional variants, molecular dynamics simulations are very useful. An important example is the work of Arnold et al.,^32^ in which molecular dynamics simulations and Markov models were used to evaluate the effect of a single mutation in a flexible loop region of the nitrating cytochrome P450 TxtE. Recently, Rosetta design^26^ and molecular dynamics simulations^15,33−35^ with scoring for near-attack conformations^36^ along the simulated trajectory^17,21,37,38^ were used to computationally design and rank enzyme variants. Near-attack conformations (NACs) are transient conformations of enzyme–substrate complexes that are close to the transition state. Their importance as a contributor to transition state stabilization and efficient catalysis is disputed,^39,40^ but in both interpretations, the frequency of occurrence of such NACs should be correlated with the relative rates of catalysis, such as those that determine stereoselectivity. Multiple short MD simulations are used to achieve sufficient throughput and better conformational sampling.^21,41^ This approach of multiple independent short MD simulations with scoring for NACs can accurately select stereoselective variants from a large number of mutant enzyme–substrate combinations generated by computational enzyme design.^17,21,37,38^ An example is the CASCO protocol, which searches for the best candidates among thousands of possibilities and provides libraries of which only 10–30 variants must be tested to find desired enzymes.^17,37,38^ Thus, by careful ranking, protein design algorithms can steer the design of small libraries, filling a gap between rational design and directed evolution.

Due to the marked flexibility in their active-site region and the occurrence of ligand-induced conformational changes,^42^ cytochrome P450s are challenging enzymes for the rationalization of catalytic properties by computational approaches.^43−45^ Recently, short MD simulations were performed on P450s to examine selectivity in the hydroxylation of steroids,^46^ warfarin,^47^ testosterone,^48^ terpene and limonene,^49^ and other substrates.^50−56^ In these examples, P450 variants were studied using nanosecond timescale MD simulations with scoring for catalytically productive conformations along MD trajectories, which explained the observed stereoselectivity. Moreover, nanosecond timescale MD on P450s was employed to report on binding properties of aflatoxin B1,^57^ caffeine,^58^ and others.^59−61^

**Figure 1:**
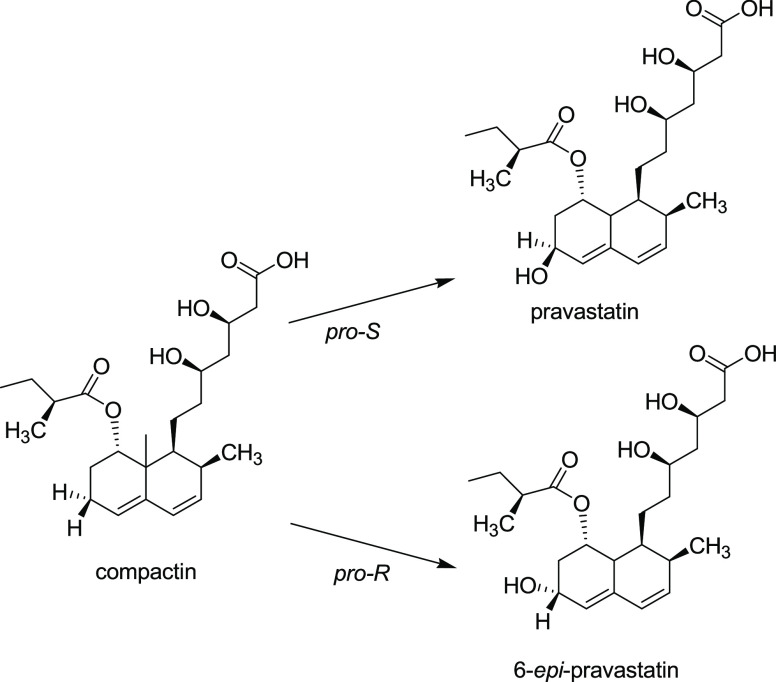
Hydroxylation of compactin by CYP105AS1 variants. The epimeric composition of the product is determined by *pro-S* vs *pro-R* stereopreference of hydrogen abstraction by the Compound I iron(IV)-oxo porphyrin radical intermediate.

The current study concerns computation-supported optimization of the stereoselectivity of CYP105AS1, a P450 from *Amycolatopsis orientalis* (Figure 1). The wild-type enzyme catalyzes the hydroxylation of compactin, a glucose-derived metabolite formed by *Penicillium citrinum*, to yield 6-epi-pravastatin. The opposite epimer, pravastatin, is an LDL cholesterol-lowering drug that is marketed as Pravachol for the treatment of hypercholesterolemia and dyslipidemia.^62^ Although pravastatin is not the most powerful statin,^63^ it is an attractive therapeutic agent because it is not significantly metabolized by human CYP3A4 or CYP3A5^64^ and shows low adverse interactions with other medications.^65^ A P450-catalyzed hydroxylation of compactin to pravastatin would enable the development of an attractive direct fermentative production process. Directed evolution has yielded derivatives of CYP105AS1 that exhibit the necessary opposite stereopreference, producing mainly pravastatin instead of 6-epi-pravastatin. However, the selectivity in this asymmetric transformation is still modest, with product epimeric excess (e.e.) not exceeding ca. 90%.^66^

In the current work, we used a computationally guided approach to create a CYP105AS1 variant for nearly perfect stereoselective production of pravastatin. Using Rosetta CoupledMoves to search sequence and conformational space around the active site, we identified substitutions predicted to contribute to the desired stereoselectivity. For verification, these mutations were introduced into the template obtained by directed evolution, which led to an increase in pravastatin purity to >99% e.e. The increased product e.e. could be explained by MD simulations with scoring for stereodiscriminating near-attack conformations of enzyme–substrate complexes. Ultrashort MD simulations (picoseconds timescale) were used to distinguish successful P450 designs, opening new opportunities for scoring of selective P450 variants at a low computational cost. The results demonstrate the use of computational approaches to predict cytochrome P450 variants with enhanced stereoselectivity.

## Materials and Methods

### Preparation of Enzyme–Substrate Complexes for Rosetta CoupledMoves

For modeling of the substrate compactin (already present in PDB 4OQR), a rotamer library was generated using the ConfGen function in Schrödinger Maestro.^67^ The bicyclic structure was rigidly constrained and redundancy was avoided by removing overlapping rotamers (RMSD < 0.2 Å). A total of 50 rotamers were selected, in which the conformation of the tail of the substrate was variable.

The P450pra crystal structure in complex with compactin (PDB 4OQR) was prepared for computational modeling using the Protein Preparation Wizard feature in Maestro.^68^ This structure is missing 5 amino acids (Gly83 to Lys87) in a loop region. The loop was modeled onto the crystal structure using Yasara,^76^ keeping the original backbone structure for the rest of the protein. The resulting structure was relaxed by energy minimization using the OPLS3 force field.^69^ In this structure, the distance between the iron of the heme and the reacting substrate hydrogen is not optimal for attack by compound I during catalysis.^70−72^ Therefore, prior to redesign calculations, the substrate was slightly repositioned manually to produce a reactive *pro-S* conformation with a distance between the reacting hydrogen and the heme iron of 4 Å, which was constrained during subsequent Rosetta optimization trajectories.

In the wild-type CYP105AS1 crystal structure (PDB 4OQS), no substrate is present and the enzyme has an open conformation. The oxygenase reaction occurs in a closed conformation that is adopted upon substrate binding.^73^ For computational modeling, we therefore created the wild-type structure from the P450pra structure, which has a closed conformation, by reverting the mutations T95I, R127Q, V180A, I236L, and N265A using PyMOL^74^ followed by relaxation of the structure as mentioned for P450pra.

Since no structures of wild type or mutants are available with compactin in a *pro-R* orientation, computational docking was used to generate a series of conformations from which a complex with substrate in the *pro-R* pose was selected. For docking, we here used the AutoDock Vina protocol^75^ included in Yasara software.^76^ Binding modes were separated in clusters with a ligand RMSD of 5 Å. The docking protocol considered protein flexibility by generating five low-energy protein structures that differed in side-chain rotamer conformations. The substrate compactin was docked 24 times for each initial enzyme conformation, giving a total of 120 individual docking solutions per trial. The best solution was selected for Rosetta design. The lowest-energy conformation served as the input structure for Rosetta CoupledMoves calculations. An additional *pro-R* starting conformation was generated by manually replacing the crystallographic *pro-S* conformation to a *pro-R* pose.

### Rosetta CoupledMoves Optimization

To define the initial sequence space to be searched in Rosetta calculations, residues within 5 Å of the substrate in the P450pra crystal structure were selected. This selection was trimmed as follows. A BLAST search on UniProt Reference Cluster UniRef90^77,78^ was used to select sequences with less than 90% identity to CYP105AS1 and, using an alignment of the 50 most similar sequences made with BioEdit,^79,80^ residues that were highly conserved were omitted from the search space. Dependent on the purpose of the calculations, the search space was occasionally further trimmed, for example, to examine the *in silico* recapitulation of the P450wt to P450pra mutations. For simultaneous dock-and-design calculations (*i.e.*, calculations in which both the position of the substrate and the protein sequence were optimized), Rosetta CoupledMoves (version 57576) was used with the following options: “-nstruct 30 -extra_res_fa COM.params HEM.params -coupled_moves::initial_repack false -coupled_moves::save_structures true -coupled_moves::mc_kt 0.6 -coupled_moves::mm_bend_weight 1.0 -coupled_moves::ntrials 10000 -coupled_moves::ligand_mode true -coupled_moves::ligand_prob 0.1 -coupled_moves::ligand_weight 2.0 -coupled_moves::fix_backbone false -coupled_moves::uniform_backrub false -coupled_moves::backbone_mover backrub -coupled_moves::bias_sampling true -coupled_moves::bump_check true -coupled_moves::trajectory true -coupled_moves::trajectory_file traj.pdb -coupled_moves::trajectory_stride 500 -coupled_moves::number_ligands 2 -use_input_sc -ex1 -ex2 -extrachi_cutoff 0”.

Initially, 14 positions (residues 76, 80, 93, 95, 179, 180, 182, 235, 236, 239, 282, 286, 388, 389) were allowed to mutate into rotamers of all 20 amino acids. The heme pdb file was converted to a MOL file type and parameterized in Rosetta. Heme and substrate were set free to move (albeit with a distance restrained, as described above). Productive binding modes were supported during docking and redesign using constraint files limiting the distance of the heme iron to the C6 carbon of compactin to <5 Å. The number of trajectories (-nstruct) was set to approximately 30, with the number of Monte Carlo sampling steps per trajectory (-ntrials) set to 10,000. Low-energy redundant sequences were trimmed to keep only unique sequences.

The most promising designs were selected based on Rosetta energy scores. Mutations altering enzyme specificity were predicted by comparing the enrichment of substitutions between large sets of Rosetta designs obtained with substrate docked in *pro-S* or *pro-R* conformations. The percentage enrichment (PE) for each mutation was calculated as before^30^ with
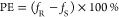
1in which *f*_R_ and *f*_S_ are the frequencies of occurrence of substitution in the set of *pro-R* and *pro-S* designs, respectively.

### Molecular Dynamics Simulations

Different variants of CYP105AS1 in complex with substrate were examined by molecular dynamics simulations. Cytochrome P450 starting structures were generated in PyMOL^74^ (*e.g.*, for creating P450pra, P450pra100 (+T95F, V180M), and the single mutants P450pra + T95F and P450pra + V180M), with additional steps described above for wild-type CYP105AS1. For P450pra, we adopted the position of the substrate as it was present in the crystal structure. For wild-type CYP105AS1, adopting the binding pose from the P450pra crystal structure resulted in clashes, but computational docking with Autodock VINA within YASARA yielded binding poses indicative of *pro-R* attack, in agreement with the observed formation of 6-epi-pravastatin. These computations were performed with 999 runs for each mutant and 25,000 energy evaluations per run. Flexibility was allowed for side chains within 4 Å from the substrate. For the P450pra100 variant, alignment of the model with the P450pra crystal structure and adopting the compactin binding mode gave a good fit in the active site, which was used for the simulations. The same substrate orientation was found to fit in the P450pra+T95F and P450pra+V180M starting structures. The compound I state was generated for all variants using the crystal structure of P450pra containing the heme cofactor.

**Figure 2:**
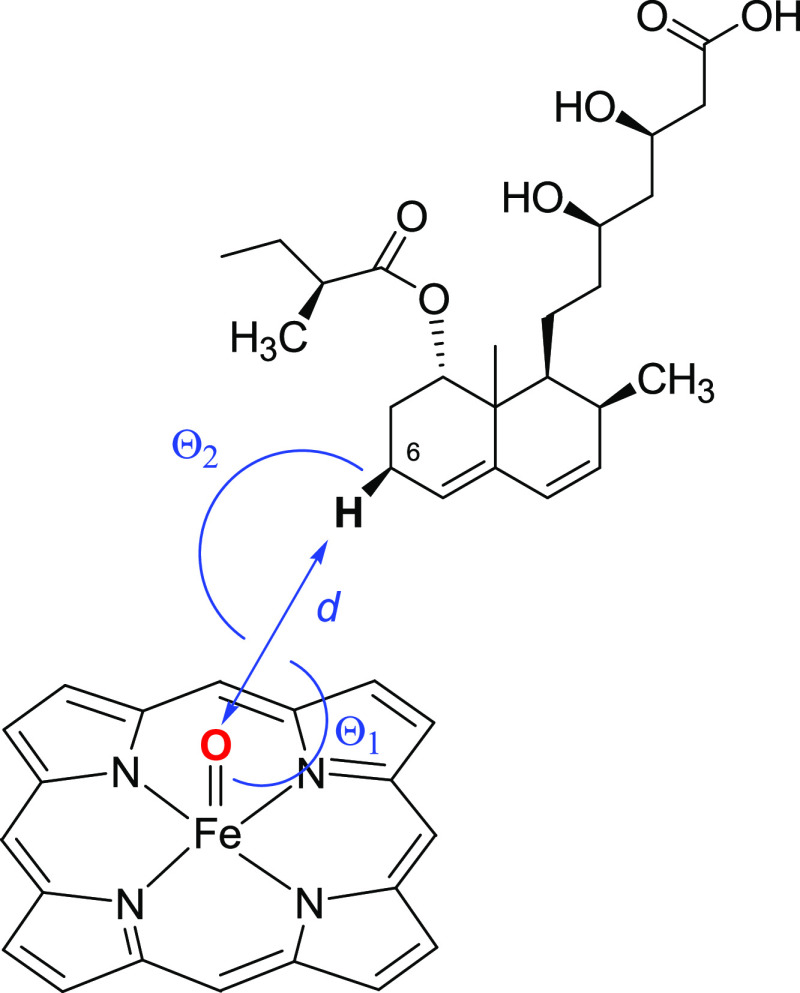
Definitions of NACs for P450-catalyzed compactin (6*S*)-hydroxylation. The symbol *d* denotes the distance between the reactive oxygen and the hydrogen (*pro-S* is shown) that is replaced by a hydroxyl group. θ_1_ is the angle between the iron, the reactive oxygen, and the hydrogen. θ_2_ is the angle between the reactive oxygen, the carbon, and the hydrogen of the substrate. NAC criteria were: *d* ≤ 2.7 Å; 100° < θ_1_ < 140°; and θ_2_ > 140°. The same criteria were used for *pro-R* NACs.

Simulations were performed using the Yamber3 force field,^81^ which is a derivative of Amber99. Runs were done with a time step of 2.0 fs and periodic boundary conditions. The atomic point charges of the substrate compactin were generated using AM1-BCC.^82^ The atomic point charges of the compound I heme were adopted from Seifert et al.^47^ Similar parameters for the compound I heme were reported by Shahrokh et al.^83^ After energy minimization and a gradual warm-up (from 5 to 298 K in 30 ps), five independent MD simulations were run for each enzyme variant. The simulations were run using different initial atom velocities, randomly chosen, but following a Maxwell–Boltzmann distribution. The system was equilibrated for 2 ns, followed by a production phase of 20 ns, and snapshots were saved every 50 ps. During the production phase, the geometry of the enzyme–substrate complex was analyzed on the fly with 1 ps intervals for the presence of NACs (Figure 2). The NAC criteria were reported previously and are based on previously reported DFT calculations on the modeled transition state.^46,84^

To test if a larger number of shorter independent MD simulations would increase conformational sampling and agreement with experiments, also 50 shorter MD runs were performed for each enzyme variant, each replica with independently assigned initial atom velocities.^17,21^ After warm-up for 30 ps and an equilibration time of 20 ps, the production phase was run for 50 ps. Snapshots were saved every 5 ps and the geometries were analyzed for NACs every 20 fs on the fly. From the simulations, the predicted epimeric excess (e.e.) was calculated with eq 2, where NAC_R_ and NAC_S_ stand for the percentage of snapshots that obey the *pro-R* and *pro-S* NAC criteria, respectively.
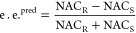
2

### Bond Dissociation Energies

To calculate the dissociation energies of different carbon–hydrogen bonds in compactin, a series of quantum mechanical calculations were performed using Gaussian09. The bond dissociation energies were obtained from the difference in formation enthalpies at 298 K of the substrate, the hydrogen radical, and the different substrate radicals.^85^ It was verified that no imaginary frequencies were present after energy minimization. The B3LYP density functional method was used with the 6–31G(d) basis set since it was shown earlier that this combination predicts bond dissociation energies with excellent correlation (*r* = 0.991) to experimental data.^86^ With a small test set (methane, ethane, ethene, propane, propene, benzene, ethylbenzene) we verified that the method indeed gave good agreement with experimental values.^85^

### Cloning, Mutagenesis, Expression, and Enzyme Isolation

The CYP105AS1 gene from *A. orientalis* (accession number KF751385) and derivatives thereof were expressed using a pBAD/HisA vector, with an N-terminal polyhistidine tag, under control of a minimal arabinose promoter (*araBAD*), from a pBAD-DEST49 donor vector using the *attr1*/*attr2* Gateway sites. Starting with the P450pra variant, mutations were created using a QuikChange mutagenesis kit (Agilent Technologies) as described.^66^ Transformed cells of *E. coli* One Shot Top10 were incubated at 37 °C with shaking at 195 rpm in 0.6–1.0 L volumes of 2 × YT broth (Formedium) with 75 μg/mL ampicillin. When cultures reached an OD600 of 0.5, 1.3 mM l-arabinose and 500 μM of δ-aminolevulinic acid were added to induce expression, and cultivation was continued at 24 °C for 16 h. Cells were harvested by centrifugation at 9000*g* and washed with ice-cold buffer A (50 mM potassium phosphate, 300 mM KCl, 10% glycerol, pH 8.0). Pellets were frozen at −20 °C. Further steps were done at 4 °C. For lysis, cells were resuspended in buffer A and Complete EDTA-free protease inhibitor cocktail (Roche, 1 tablet per 50 mL of cell suspension) and 100 μg/mL DNAse I (Sigma-Aldrich) were added. Cells were lysed using an MC cell disruptor at 17.5 kPSI and the homogenate was cleared by centrifugation at 70,000*g* for 60 min.

For enzyme isolation, the clear lysate was incubated overnight with Ni-IDA-Metal Chelate Sepharose resin (Serva) with 15 mM imidazole and with mixing. After washing with 20 volumes of 50 mM Tris, pH 7.2, containing 20 mM imidazole, protein was eluted with 5 volumes of buffer B (50 mM Tris, 1 mM EDTA, pH 7.2) containing 250 mM imidazole. The protein was dialyzed with buffer B to remove imidazole and further purified on a 6 mL Resource Q anion exchange column, with elution by buffer B with 0–400 mM KCl. After dialysis and concentration by Amicon ultrafiltration (0.2 μm membrane), highly pure proteins were obtained.

### Expression and Purification of FldA and FldR

Cells were grown according to the published method,^87,88^ and purification was done as with the following adaptations.

Enzymes were purified using a HiPrep Q XL 10/16 anion exchange column (Cytiva), with elution by 15–20 column volumes of buffer containing 0–1 M KCl. After removal of salt using a PD-10 desalting column (Cytiva), FldR was further purified using a 6 mL Resource Q anion exchange column (Cytiva) with elution by 20 volumes of buffer B containing 0–1 M KCl. The purified proteins were desalted using a PD-10 column and concentrated using a Vivaspin 20 MWCO centrifugal concentrator (Sartorius), flash-frozen, and stored at −80 °C.

### UV–Vis Spectroscopy

Absorbance spectra (300–800 nm) of P450 variants were measured on a Cary 60 UV–vis spectrophotometer at 25 °C using 3–8 μM protein in buffer A. The pyridine hemochromogen method was used to quantify CYP105AS1 P450 variant heme concentrations and to determine an extinction coefficient at the heme Soret maximum for each variant.^89^ For titration, compactin (0.05–0.5 μL) was added from 5 to 50 mM stock solutions in DMSO. The overall absorbance change (Δ*A*_max_) was calculated as *A*_peak_ (absorbance maximum) minus *A*_trough_ (absorbance minimum) and plotted against the substrate concentration. Data were fitted according to a standard hyperbolic binding equation or a Morrison equation for tight-binding to determine dissociation constants (*K*_d_).^90,91^

### Enzyme Turnover Reactions

Reactions were done in 0.5 mL volumes containing 1 μM of enzyme, 10 μM FldA, 2 μM FldR, and 20 μM compactin in buffer D (50 mM potassium phosphate, pH 7.4). Reaction mixtures also contained an NADPH regeneration system composed of 600 μM NADP, 7.76 mM glucose-6-phosphate, and 0.75 U/mL glucose-6-phosphate dehydrogenase from *S. cerevisiae* (Sigma-Aldrich). After adding the P450 enzyme, incubations were continued for 2 h at 37 °C with shaking at 195 rpm. Reactions were stopped and proteins were precipitated with acetonitrile for 20 min. After extraction of the sample under vacuum by mixing with Strata-X 33 μm Polymeric Reverse Phase solid-phase extraction material (Phenomenex), the sample was eluted with 0.5 mL of methanol and transferred to glass vials for analysis.

For large-scale reactions, the same components were used in a final volume of 60 mL and a P450 protein concentration of 0.5 μM. FldA was added at 5 μM and FldR at 1 μM. NADPH and compactin concentrations were as above. After extraction with Strata-X 33 μm Polymeric Reverse Phase solid-phase extraction polymer (sorbent mass/volume of 10 mg/mL), the sample was eluted with 0.5 mL of methanol and used for analysis.

### HPLC and LC-MS Analysis

Samples (40 μL) were analyzed by high-performance liquid chromatography (HPLC) on an Agilent 1100 Series instrument equipped with a Kinetex Evo C18 ultraperformance UPLC column (Phenomenex, 50 mm × 4.6 mm, particle size 2.6 μm). Isocratic elution was done with buffer E (60% H_2_O, 20% methanol, 15% acetonitrile, 5% tetrahydrofuran, and 0.1% formic acid) at 0.4 mL/min. Any residual components were eluted using a 0–95% gradient of acetonitrile. Detection was at 238 nm (for substrate and products) or at 280 nm (for other components).

Peaks were identified by LC/MS. For this, samples (2 μL) were separated on a Kinetex EVO C18 UPLC column (Phenomenex) operated isocratically with buffer E (60% H_2_O, 20% methanol, 1% acetonitrile, 5% tetrahydrofuran and 0.1% formic acid) and connected to an electrospray ionization (ESI) mass spectrometer on an Agilent 6470 Triple Quadrupole (Triple-Q) LC/MS (Agilent, Cheadle UK). For product determination, mass/charge ratio values (*m*/*z*) for precursor ions of products were monitored. The pravastatin precursor ion was identified by selective ion monitoring with a *m*/*z* of 432.3 using a fragmentor voltage of 70 V. The product ion was detected with a *m*/*z* of 321.1 using a collision energy of 14 V.

## Results

### Rosetta Design Reproduces Mutations Found by Directed Evolution

Wild-type CYP105AS1 catalyzes the hydroxylation of compactin to 6-epi-pravastatin, the unwanted 6-epimer of the pharmaceutically active compound pravastatin. Using error-prone PCR and screening, McLean et al.^66^ obtained a variant that carries 3 substitutions in the active site (I95T, A180V, L236I) and 2 mutations on the surface (Q127R, A265N). This variant (P450pra) showed both a 21-fold higher affinity for compactin and a switch in stereoselectivity, producing mainly pravastatin, which is hydroxycompactin carrying the hydroxyl group in the 6*S*-position (Figure 1). To explore the use of computational protocols for enhancing the desired 6*S*-stereoselectivity further, we first examined if Rosetta CoupledMoves would rediscover the beneficial active-site mutations of P450pra.

Since the crystal structure of the wild-type CYP105AS1 (PDB ID: 4OQS) is in an open state, the wild-type enzyme was modeled by reverting the mutations in the P450pra crystal structure (PDB ID: 4OQR). Subsequently, the substrate compactin was docked in the active site in a *pro-S* binding mode, and its position was partially constrained during design calculations.^37^ Initial Rosetta runs were done allowing certain active site positions of CYP105AS1, namely, those that changed in the directed evolution variant (95, 180, and 236), to mutate to all 20 proteinogenic amino acids. With these settings, the active site was redesigned using 26 trajectories of each 10,000 iterative steps of Monte Carlo sampling in Rosetta CoupledMoves. CoupledMoves was earlier tested with 20 trajectories of just 1000 iterative steps, which was sufficient for reproducing beneficial mutations in other enzymes.^30^ The current redesign produced 1748 low-energy solutions for the identities and rotamers of the amino acids at the 3 positions.^30^ This set was reduced to 251 unique low-energy designs by eliminating high-energy copies of identical sequences. The A180I and L236I mutations of P450pra were found with high frequencies among this set of designs (Table S1, Figure S1A). Mutation I95T of P450pra was the fourth most common mutation at position 95 found by Rosetta, after I95W, I95F, and I95V. From the Rosetta energy scores, the sequence of P450pra was predicted as the 8th most favorable design for improved binding of compactin (Table S2). The compactin molecule was bound in these designed variants in the same position as in the P450pra crystal structure which is the desired *pro-S* orientation. This result suggested that the Rosetta CoupledMoves protocol can accurately reproduce a catalytically active sequence and model its structure, the latter including the position of bound substrate and the conformation of mutated residues (Figure S2).

### Selecting the Rosetta Design Search Space

To use Rosetta CoupledMoves to predict mutations that further improve the stereoselectivity of P450pra, we broadened the search space on the basis of structural and phylogenetic information. The P450pra sequence was aligned with similar sequences retrieved by a Blast search of the UniProt database to identify positions around the active site that show variation that may influence selectivity. Based on the diversity of the aligned sequences and active site topology, 14 positions (F76, P80, W93, I/T95 (mutated to T in P450pra), M179, A/V180 (V in P450pra), V182, L235, L/I236 (I in P450pra), V239, V282, T286, A388, and A389) were selected to be included in Rosetta calculations. This set excludes some highly conserved residues to avoid interference with essential functions, such as residue T244 and heme-binding residues. The template structure was based on the crystal structure of P450pra. Calculations included compactin bound in the *pro-S* orientation.

With all 20 amino acids allowed at these 14 selected positions, and using 30 different trajectories of 10,000 iterative steps each, Rosetta generated 12,569 low-energy designs binding substrate in a *pro-S* conformation, which was reduced to 9052 unique sequences by removing high-energy duplicates. The frequency of occurrence of each amino acid at each position in this virtual library was calculated (Table S3). At six positions (W93, L235, I236, V239, V282, and A389), most designs maintained the P450pra residue or introduced an amino acid with very similar properties. For example, position W93 kept the wild-type Trp in the majority of the low-energy designs and also I236 (mutation of P450pra) and A389 were maintained. Since mutations at these 6 positions had no significant effect on the position of the compactin in the predicted structures, they were omitted from the search space in the subsequent optimization steps.

At the other eight positions included in the Rosetta search, the resulting mutations showed structural credibility. The diversity among design solutions was again relatively moderate (Table S3). For example, residue Pro80 was frequently mutated to Ala or Phe, depending on the environment. The occurrence of Phe80 always coincided with a smaller residue at position 179, *e.g*. Ala, whereas an Ala at position 80 was most often accompanied by the larger Met at position 179. Inspection of the predicted structures showed that these combinations allowed filling of space in the active site, indicating that positions 80 and 179 should be included in further calculations aimed at optimization of selectivity. Position 95 showed significant diversity in the 9052-member virtual library, with the P450pra mutation I95T occurring in only a few of the low-energy sequences, while Val, Trp, and Phe were found more often. Inspection of the structure suggested that Trp and Phe at position 95 can fill a large void in the active site and stabilize bound compactin. Positions T286 and A388 also tolerated a large diversity, allowing substitution by both smaller or larger residues or even charged amino acids. Analysis of the structures suggested interaction between these two positions, only one of them being aromatic in the low-energy solutions. Finally, position 180 mostly kept the Val of P450pra, with some probability of replacement by a Thr or Met (Table S3). In particular, the rotamer configuration of a Met at position 180 seemed to protrude the side chain deeply into the active site, hindering undesired *pro-R* binding modes. Because at these eight positions the computational design produced mutations that showed structural credibility, these positions were included in a subsequent focused Rosetta CoupledMoves optimization step.

### Rosetta Optimization of Asymmetric Conversion of Compactin to Pravastatin Conversion

**Figure 3:**
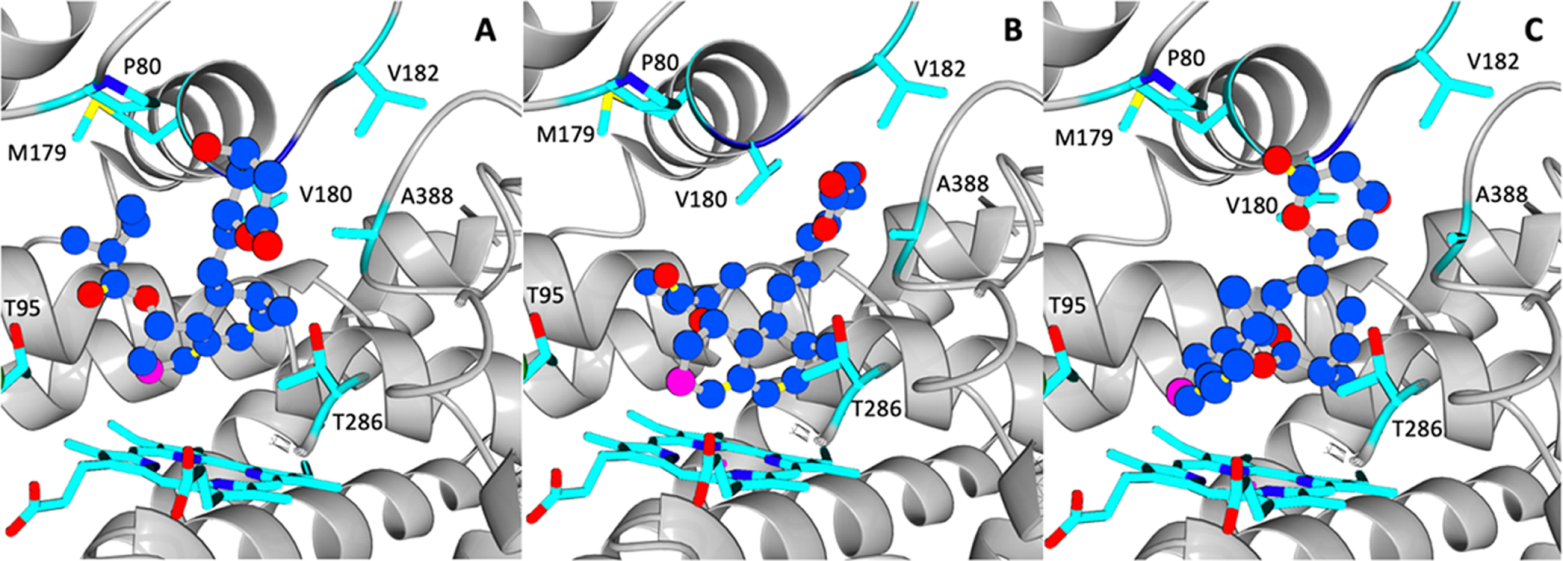
Compactin initial binding poses for Rosetta calculations. (A) Compactin manually docked in a desired pro-*S* binding mode; (B) manually docked compactin in a *pro-R* binding mode (Prava-epi1); (C) compactin in a *pro-R* binding mode compactin generated by AutoDock Vina calculations (Prava-epi2). The carbon atom that is hydroxylated is shown in fuchsia. The lactone is in the closed form, as in the crystal structure.

After computational discovery of the eight most promising target positions, Rosetta CoupledMoves was used to identify mutations that contribute to selectively binding compactin in a pravastatin-producing *pro-S* mode. In an earlier study on the computational design of epoxide hydrolase, we found that this approach can identify specific substitutions contributing to enantioselectivity.^17^ The eight positions selected above (76, 80, 95, 179, 180, 182, 286, and 388) were targeted with sampling of all 20 amino acids. Parallel optimization runs were performed with compactin constrained in a *pro-S* and in a *pro-R* conformation. The template structure was prepared from P450pra. The *pro-S* reactive conformation was obtained by slight repositioning of the substrate found in the crystal structure of P450pra (Figure 3A) and *pro-R* conformations were generated by Autodock VINA computational docking (Figure 3B). The optimization runs were done using 30 Rosetta trajectories with 10,000 iterative steps of Monte Carlo optimization to sample diversity.

Calculations with compactin in the desired *pro-S* binding mode gave 7155 low-energy designs which were trimmed to 3417 unique sequences by removing the higher-energy designs with the same sequence. The remaining set included the P450pra amino acids Phe76/Pro80/Thr95/Met179/Val180/Val182/Thr286/Ala388 at rank 1494 based on the Rosetta energy function, while no CYP105AS1 wild-type sequence was found. The five most frequent mutations are given in Table S4. In this set, Phe at position F76 was often replaced by another large hydrophobic residue. The expected favorable mutations T95F and T95W were present among the subset of the top 200 design solutions with the lowest Rosetta energy, but V180M was not (Figure S1B). Based on the above observation that Met180 seems to disturb *pro-R* binding of compactin, we considered that V180M improves stereospecificity without contributing to the lowest overall energy. Combinations of Ala80 and Met179 were again observed, confirming an epistatic effect at these positions. Amino acids introduced at positions V182, T286, and A389 showed high diversity in hydrophobicity, charge, and size.

The parallel CoupledMoves calculations with compactin in the undesired *pro-R* binding mode were done both with the structure obtained by manual repositioning of the substrate (Prava-epi1, Figure 3B) and with an enzyme–substrate complex obtained by Autodock Vina (Prava-epi2) (Figure 3C). These calculations generated 6048 and 6892 low-energy *pro-R* designs with unique sequences, respectively. The most common amino acids at positions 95 and 180 in both sets were the wild-type CYP105AS1 Ile and Ala, respectively (Table S5), suggesting that these amino acids contribute to the unwanted 6-epi-pravastatin production in the wild type and that substitutions contributing to the desired stereoselectivity can be identified by their relative frequency of occurrence in Rosetta CoupledMoves designs.

The role of mutations at positions 95 and 180 was further examined by combining the two sets of mutations in 6-epi-pravastatin designs and comparing them to amino acids found in pravastatin designs (Table 1). This confirmed the clear preference for the wild-type Ile at position 95 and Ala at position 180 among the 6-epi-pravastatin designs, whereas pravastatin designs preferably harbored a Val or Phe at position 180. A Met was present at a low frequency among the latter designs, but missing among *pro-R* designs. Similarly, a Phe was only found at position 95 among *pro-S* designs, suggesting mutation T95F also contributes to the desired stereoselectivity. Another residue potentially influencing stereoselectivity was at position 286, with a strong preference for Phe in designs optimized for pravastatin binding.

**Table 1:** Comparative Amino Acid Enrichment for Pravastatin and 6-epi-Pravastatin among Rosetta CoupledMoves Designs^a^

	pro-pravastatin		pro-6-epi-pravastatin	
position	preferred AA	comparative enrichment (%)	preferred AA	comparative enrichment (%)
P80	A	28.49	G	13.88
T95	V	31.44	I (wild-type)	67.81
M179	M (wild-type)	34.17	L	27.04
V180	V (P450pra)	44.91	A (wild-type)	22.87
V182	V (wild-type)	12.08	A	11.14
T286	F	28.53	L	39.92
A388	A (wild-type)	18.71	N/A	N/A

a The differential enrichment of substitutions among Rosetta designs optimized for compactin binding in pro-pravastatin versus pro-6-epi-pravastatin binding mode were calculated. Values represent differences in amino acid abundance in the pravastatin and 6-epi-pravastatin set (eq 1).

To further confirm the role of the selected residues in stereo discrimination, Yasara docking simulations were done with four low-energy Rosetta designs (named PD0001, PD0060, PD0175, and PD0254) containing mutations that were expected to support the desired pro-pravastatin binding mode (Table 2, Figure S3). The resulting enzyme–substrate conformations were clustered based on ligand heavy atoms (RMSD < 5 Å). The conformations of the lowest-energy clusters were selected as the most likely substrate binding orientations (Table 2). The predicted compactin binding energy for PD0001, which also harbored the T95V mutation supposedly not supporting *pro-S* selectivity, was similar to that for P450pra, while stronger binding was predicted for PD060, PD0175, and especially PD0254. With PD0254, the *pro-S*-favoring residue Met180 adopted the same rotamer conformation in the structure generated by AutoDock Vina and in the structure predicted by Rosetta CoupledMoves. In both cases, the Met side chain points into the active site, favorable for compaction binding in the *pro-S* mode. The side chains introduced by mutations T95W and T95F in PD0175 and PD0254 also had the same conformations in the docked structures and the Rosetta designs. Mutation T95V was present among both *pro-S* and *pro-R* designs (Tables S4 and S5), suggesting it will not improve the selectivity for pravastatin yet improve compactin binding. The predicted ability of T95W, T95F, and V180M to improve binding, and their repeated occurrence with similar side-chain rotamers found in different designed structures, suggested these substitutions could play a key role in shaping the binding pocket and in improving stereo discrimination.

**Table 2:** Predicted Properties of Four Compactin Designs^a^

design	P450pra	PD0001	PD0060	PD0175	PD0254
residues at positions 76, 80, **95**, 179, **180**, 182, 286, 388	FP**T**M**V**VTA	FA**V**M**V**VVA	FA**F**M**T**VNA	VA**W**M**V**VMA	FFVG**M**TWG
Rosetta energy score	132.6	132.6	130.9	132.2	128.2
Yasara-predicted binding energy (kcal/mol)	9.5	9.5	10.3	10.3	10.4
predicted dissociation constant (μM)	110	110	28.9	27.9	23.8

a The four designs represent low-energy sequences with conformations selected by docking simulations with Rosetta, optimized for pravastatin production.

Based on these docking results, the visual inspection and the discriminatory contribution of mutations to stereoselectivity suggested by the Rosetta calculations, mutations at positions F76, P80, T95, V180, and T286 were selected for introduction in the P450pra template, which already carries L236I (Table 3). This set of mutations thus results from an integrated approach, where Rosetta identified the most influential positions and beneficial mutations, and docking simulations and visual inspection served to pick mutations which contributed most to the desired stereoselectivity. The use of visual inspection to select for experimental characterization only the most promising variants among a set of computationally designed and ranked mutants is a useful step in computational enzyme redesign.^92^ Mutations T95F and T95W were expected to be superior to T95V and T95I (reverting to the wild-type Ile95) since the former mutations were exclusively detected in *pro-S* designs (Tables S4 and S5). Furthermore, V180M contributes to *pro-S* selectivity and appears compatible with T95F in Rosetta designs. Also, after testing the single mutants, the double mutant and V180M P450pra + T95F + V180M were selected because Rosetta-designed structures indicated these substitutions are compatible and would restrict the conformational freedom of compactin bound in the active site to *pro-S* poses. Furthermore, independently from the computational approach, we tested the mutations F76N and T286I, which may introduce or remove a polar interaction with the carbonyl oxygen of the lactone ring, and P80G, which would influence the flexibility of the 76–80 loop covering the active site. These can be regarded as controls since they were not selectively enriched in the Rosetta *pro-S* designs (Tables S4−S5).

**Table 3:** Compactin Binding and Hydroxylation by P450 Variants

variant^a^	source	pravastatin^b^ (%)	6-epi-pravastatin^a^ (%)	*K*_d_ (μM)	LS/HS^c^ conversion (%)
CYP105AS1	wild type	11	89	30.3 ± 5.2	<5
P450pra^c^	error-prone PCR	95	5	0.77 ± 0.07	70–80
P450pra + F76N	rational	85	15	4.81 ± 0.25	60–70
P450pra + P80G	rational	68	32	5.84 ± 0.34	70–80
P450pra + T286I	rational	71	29	4.93 ± 0.25	60–70
P450pra + T95W	computational	79	21	3.29 ± 0.03	60–70
P450pra + T95F	computational	95	5	0.25 ± 0.01	>95
P450pra +V180I	rational	93	7	1.64 ± 0.13	80–90
P450pra +V180M	computational	97	3	1.34 ± 0.06	>90
P450pra + T95F/V180M (=P450pra100)	computational/rational	>99	<1	0.13 ± 0.03	>95

a In comparison to wild type, P450pra carries the active site mutations I95T, A180V, and L236I, and on the surface (Q127R, A265N). The mutations above are on top of these mutations.

b Percentage of total detected hydroxylation product.

c Partial low-spin (LS, absorption at 418 nm) to high-spin (HS, at 390 nm) conversion was observed upon titration with compactin.

### Experimental Verification

Variants harboring the thus selected mutations were constructed by QuikChange mutagenesis, starting from the P450pra variant. After production in *E. coli* and purification by affinity and anion exchange chromatography, pure monooxygenases were obtained and used for activity measurements and determination of substrate dissociation constants. Reaction mixtures included a flavodoxin-based electron transfer system and NADPH regeneration with glucose-6-phosphate dehydrogenase. Product compositions in terms of ratios of pravastatin epimers were calculated from peak areas determined by LC-MS (Table 3, Figures S4–S6). As expected, the wild-type CYP105AS1 produced mainly the unwanted 6-epi-pravastatin. In the case of P450pra, the product mixture contained mainly the desired pravastatin, in agreement with previously reported results. Gratifyingly, three variants displayed pravastatin production similar to, or even better than, P450pra (Table 3). Among the single mutants, the best result was found with variant V180M. All variants also carried the beneficial mutation L236I from the P450pra template. An even higher percentage of the desired epimer was found with a combination of two mutations (T95F + V180M) which gave production of pravastatin only, with no detectable (<0.5%) formation of the unwanted 6-epi-pravastatin (Table 3). We termed this mutant P450pra100 (see Figure S7 for all of its mutations) since it showed the desired stereoselectivity and was completely devoid of detectable side-product formation (Figure S6).

The observation that variants with introduced aromatic groups at position T95 gave improved production of pravastatin confirmed that this position is of key importance for the stereoselectivity switch. However, mutations at position 95 only did not sufficiently increase the epimeric ratio. Similarly, mutation V180I had a positive effect on the pravastatin to 6-epi-pravastatin ratio, but not beyond what was observed previously (Table 3). The mutation V180M gave improved production of pravastatin to 97% (*e.e.* 94%), confirming the importance of position 180. These Rosetta-predicted variants performed better than the rationally selected P450pra + F76N, which was found to produce ca. 85% of the desired epimer (*e.e*. 70%), so without improvement over P450pra. Similarly, P450pra + P80G and P450pra + T286I both produced the desired pravastatin as the dominant epimer, but at a slightly lower excess in comparison to the parent P450pra.

The observation that the combination of mutations in P450pra100 gave perfect production of the desired epimer (selectivity > 99%) indicates an additive effect of the T95F and V180M mutations. Inspection of the Rosetta-predicted structures (PD0001, PD0060, PD0175, and PD0254) showed that residues 95 and 180 occupy positions on opposite faces around the compactin substrate, suggesting a structural explanation. The combination of the confirmed beneficial substitutions at these positions in mutant P450pra100 thus gave ideal selectivity with the formation of the desired pravastatin product only. The introduction of two large side chains by the T95F + V180M substitutions influences the shape and size of the substrate-binding pocket, which we suggest leads to reduced access of substrate to undesirable reactive *pro-R* poses.

### Compactin Binding Affinity

UV–vis substrate binding studies were conducted to investigate the effect of mutations on compactin binding. All variants showed a substrate-binding-induced type-I Soret shift from a longer wavelength low-spin (LS) to a shorter-wavelength high-spin (HS) form of the heme iron (Figures S8 and S9). Titration experiments showed that the wild-type CYP105AS1 displayed a relatively poor affinity for compactin (Table 3). The P450pra variant obtained by directed evolution showed a higher affinity, whereas the rationally designed variants and Rosetta-inspired single mutants showed varying *K*_d_ values, including a 3-fold higher affinity obtained with the T95F mutation. Furthermore, the P450pra100, which gave epimerically pure pravastatin, demonstrated a stronger improvement in compactin binding and full conversion to the high-spin heme at substrate saturation. Probably, the bulky aromatic side chain of the introduced Phe95 residue influenced the binding of compactin, which allowed elimination of the proximal water ligand and full conversion to the high-spin state upon binding of this substrate.

### Molecular Dynamics Simulations

In line with the hypothesis that preferential production of pravastatin or 6-epi-pravastatin is related to differences in the occurrence of reactive *pro-S* or *pro-R* binding conformations, we investigated if epimeric ratios can be predicted by MD simulations. Since complete conformational sampling of proteins by MD simulation is impossible even at time scales of a ms,^93^ short protocols with restricted sampling are needed if MD is to be used as a ranking tool for sets of designs. Such short simulations can still reveal the formation and stability of reactive conformations of enzyme–substrate complexes. For example, scoring the frequency of occurrence of reactive conformations enabled the prediction of the regioselectivity of the hydrolase-mediated asymmetric conversion of *meso* epoxides.^21,37^

According to the P450 catalytic mechanism, the reactive oxyferryl intermediate (compound I) stereoselectively abstracts a hydrogen from the substrate, followed by oxygen rebound with retention of stereo-configuration and formation of the hydroxylated product. Therefore, the stereospecificity of hydrogen abstraction from the substrate carbon approaching the reactive oxygen was used to define *pro-R* and *pro-S* reactive conformations (near-attack conformations, NACs) (Figure 2). The frequency of occurrence (% of frames) of these conformations was recorded along MD simulations, from which stereoselectivities and e.e. values were calculated (eq 2). We used short simulation times since only access to NACs was tested, starting with substrate docked in a low-energy pose close to a reactive conformation. Further, we analyzed five trajectories with independent initialization for each enzyme–substrate complex for a total of 100 ns production run, as described in Material and Methods.

MD simulations were performed on these five enzyme–substrate complexes (wild-type CYP105AS1, P450pra, and P450pra100, P450pra + T95F, P450pra + V180M). For P450pra and further mutants, the initial position of the substrate was obtained from the P450pra substrate-bound crystal structure. This gave a snug fit in the active site, which was adopted for the simulations. In the case of wild-type CYP105AS1, adopting the binding pose from the P450pra crystal structure resulted in clashes, and therefore the substrate was positioned by docking calculations with Autodock VINA. This gave a binding pose close to a *pro-R* attack (plotted in Figure 3C). We assumed that these starting conformations can swiftly move to *pro-R* or *pro-S* NACs or to less reactive poses since only minor repositioning is needed, which was confirmed by the occurrence of *pro-R* or *pro-S* NACs but also drifts away from reactive poses in all simulations. Initially, five independent 22 ns simulations were run for each variant, which showed that the structures were stable (Figure S10). The RMSFs of three main enzyme–substrate complexes (wild-type CYP105AS1, P450pra, and P450pra100) revealed a high flexibility in the modeled loop region formed by residues 80–90 (Figure S11). This agrees with the high local flexibility suggested by the P450wt and P450pra crystal structures, in which residues are not resolved from positions V78 to G84, and G83 to K87, respectively.

**Figure 4:**
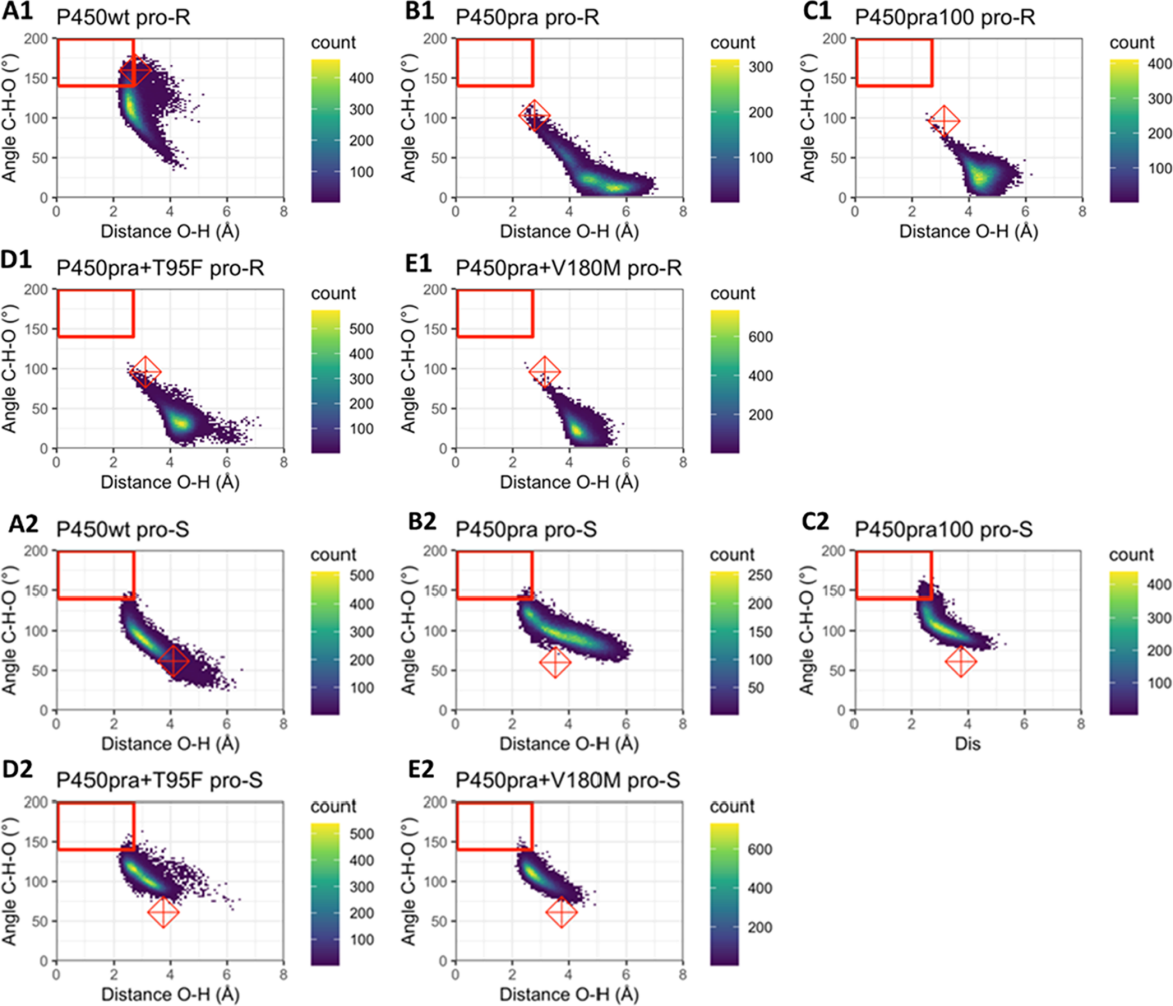
Occurrence of reactive conformations of P450:compactin complexes during MD simulations. The heat maps show C–H–O angles (θ_2_ in Figure 2) and O–H distances (*d*) along 22 ns trajectories. For each enzyme–substrate complex, five MD simulations with independent seeds were used. Distances and angles were sampled with 1 ps intervals. Colors from blue to yellow show low to high numbers of frames for each substrate orientation. The red boxes indicate orientations obeying NAC criteria for both of the shown geometric criteria (*d* ≤ 2.7 Å; θ_2_ > 140°). Note that for scoring a conformation as a NAC, also the geometric criteria for the θ_1_ angle need to be fulfilled. Therefore, not all conformations inside the red boxes are scored as a NAC. The red diamonds show angles and distances of the initial poses. (A1–E1) Plotted angles and distances relative to the *pro-R* hydrogen (whose abstraction would produce the undesired 6-epi-pravastatin); (A2–E2) corresponding distances and angles for the *pro-S* hydrogen (abstraction would produce the desired pravastatin epimer); (A) wild-type CYP105AS1; (B) P450pra; (C) P450pra100; (D) P450pra + T95F; (E) P450pra + V180M.

During MD simulations of the wild type, the starting pose relaxed mostly to catalytically less productive orientations but still retained a significant fraction of *pro-R* NACs (Figure 4, Table S6), in agreement with the observed formation of unwanted 6-epi-pravastatin. This dwelling in *pro-R* NAC poses was not observed with P450pra or other variants that mainly produce the desired pravastatin (Table S6, Figure 4A1–E1). For all of these variants *pro-S* NACs were dominant, even though none of their starting structures was near a *pro-S* NAC (Figure 4B2–E2). The stereoselectivities calculated from the frequency of occurrence of the respective NACs (e.e.^pred^) agreed with experimental results (e.e.^exp^) in all cases (Table S6). Thus, the observed stereopreference could be reproduced by the MD simulations for all five variants.

**Figure 5:**
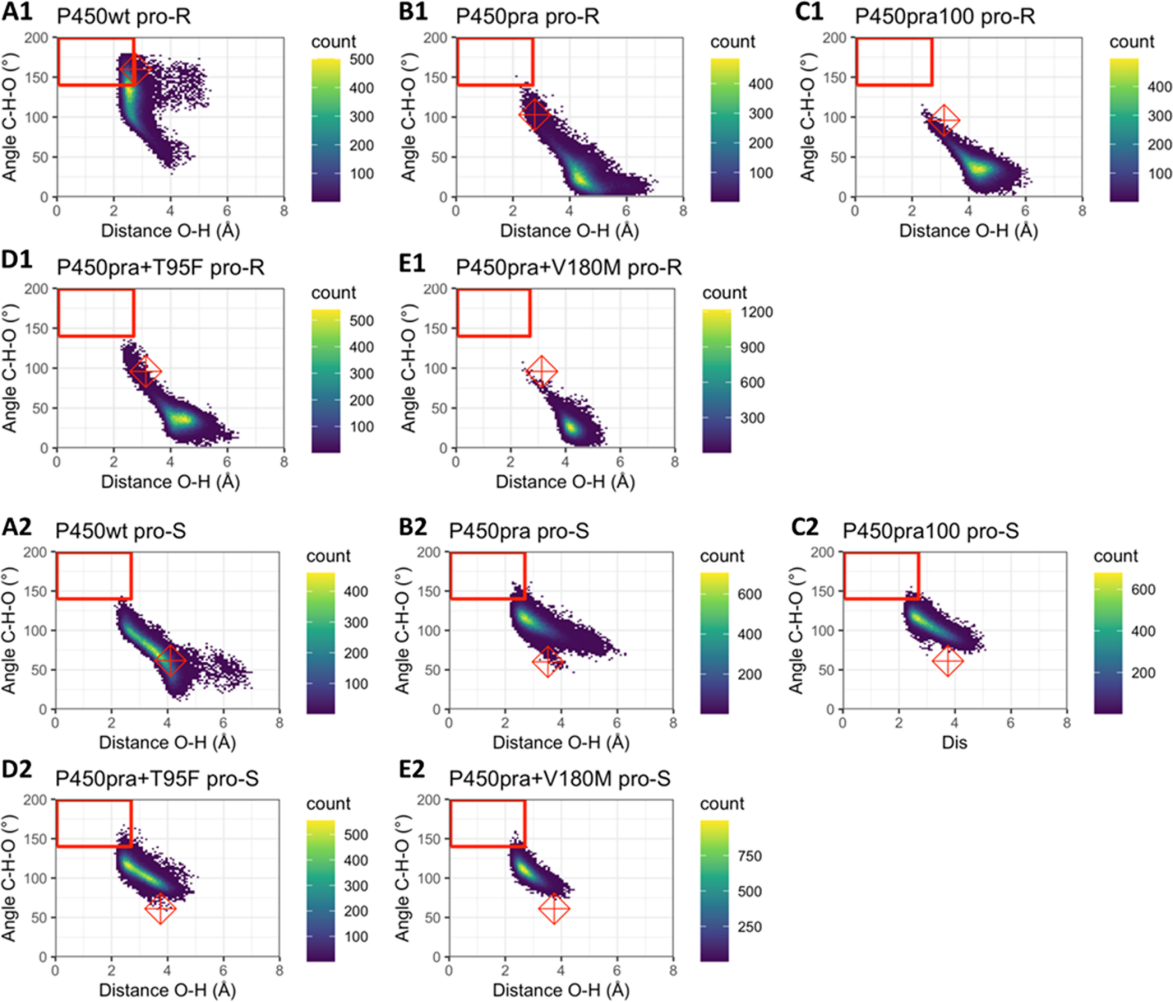
Heat map with angles C–H–O and distances O–H for ultrashort MDs. The heat maps show the C–H–O angles (θ_2_) and O–H distances (*d*) along 50 independently initiated 100 ps trajectories for each enzyme–substrate complex. Distances and angles were sampled with 20 fs intervals. Colors from blue to yellow show low to high numbers of frames for each substrate orientation. Conformations in the red boxes are considered NAC orientations (*d* ≤ 2.7 Å; θ_2_ > 140°). Please note though that for a conformation to be in NAC, also the geometric criteria for the θ_1_ angle need to be fulfilled. Therefore, not all conformations inside the red boxes are indeed in a NAC. The red diamonds show the coordinates of the initial docked pose. (A1–E1) plotted angles and distances relative to the *pro-R* hydrogen (whose abstraction would produce the undesired 6-epi-pravastatin); (A2–E2) the corresponding distances and angles for the *pro-S* hydrogen, whose abstraction would produce the desired pravastatin epimer; (A) wild-type CYP105AS1; (B) P450pra; (C) P450pra100; (D) P450pra + T95F; (E) P450pra + V180M.

The MD trajectories indicated that compactin predominantly occupied poses in which either the allylic *pro-R* or *pro-S* hydrogen is exposed to the reactive heme oxygen, but NAC conformations also occurred for several alkylic and vinylic hydrogens of the compactin bicyclic moiety (Table S7, Figure S12). However, these hydrogens have bond dissociation enthalpies that are typically 20 kcal/mol and 10 kcal/mol higher than allylic hydrogens^85^ and should thus be less reactive (Figure S12). To investigate the possibility that lower chemical reactivity of these bonds can explain the lack of reaction despite the presence of catalytically productive conformations, the dissociation energies of the compactin C-H bonds were calculated using Gaussian with a [B3LYP/6–31G(d)] function that was shown to give reliable predictions for a large variety of compounds.^86,94^ As expected, the bond dissociation energies for the alkylic and vinylic hydrogens in compactin are much higher (6–25 kcal/mol), predicting far lower reactivity than for the hydrogens at the allylic C6 position that were hydroxylated (Table S6).

**Figure 6:**
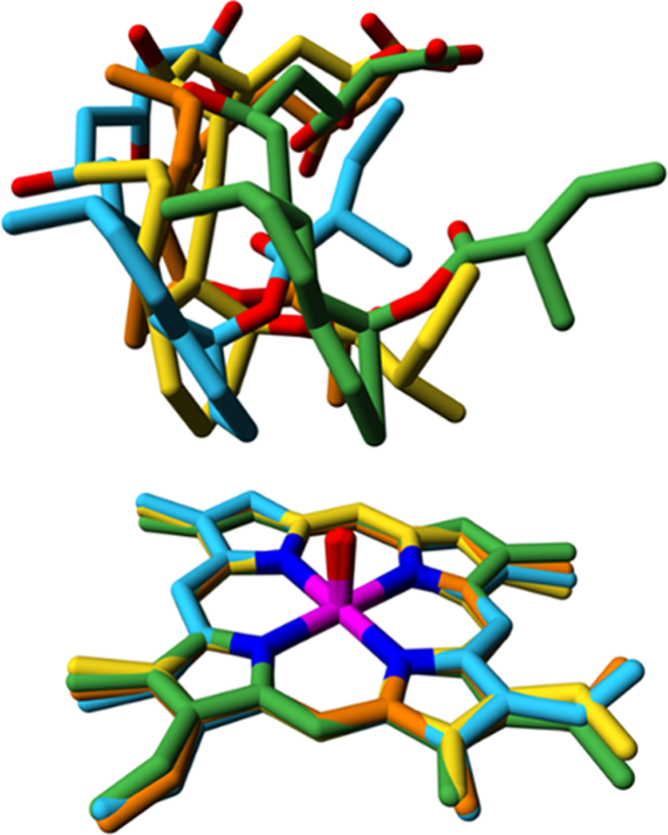
Exploration of catalytically relevant conformational diversity during ps timescale MD simulations. Conformations were sampled during 50 trajectories of 100 ps of WT CYP105AS1. Shown with yellow carbon atoms is the docked orientation of the substrate, which was the starting point of each individual trajectory. The three structurally most diverse snapshots are shown with orange, green, and cyan carbon atoms. For clarity, only the substrate and part of the heme are shown. See Figure S13 for stereo pictures, the corresponding figures for the 22 ns MD simulations, and for more details.

We also studied if shorter MD simulations can produce accurate predictions at lower computational cost per variant (Figure 5). This would enable the examination of larger sets of designs by MD screening. The use of shorter MD simulations introduces a risk that the simulations become too short to find all catalytically relevant conformations and instead only sample conformations close to the initially docked conformation. Inspection of the MD trajectories, however, indicated that the conformations sampled during 50 independently initiated 100 ps MD simulations differed at least as much from the initial docked substrate conformation as the conformations obtained from the corresponding 5 × 22 ns MD simulations (Figures 6 and S13). Furthermore, the catalytic distances and angles differ most significantly between the *pro-R* and *pro-S* hydrogens but there are also clear differences between individual trajectories (Figures S14–S19). This holds both for 100 ps and 22 ns trajectories. This all agrees with the established idea that a larger number of trajectories gives better conformational sampling than single much longer MD simulations.^41^

The protocol employing shorter but a larger number of trajectories also resulted in better agreement with experimental results because it more often sampled the relatively rare *pro-R* NACs of the mutants (Table S9). Only predictions for the wild-type enzyme still seriously overestimated its selectivity since only *pro-R* NACs, belonging to the 89% of 6-epi-pravastatin that it produces, were found, whereas experimentally it also produces 11% pravastatin (Table 3). For P450pra100 and P450pra + V180M, there were only *pro-S* NACs, in satisfactory agreement with their excellent to perfect epimeric selectivities (97, >99%). For P450pra (selectivity 95% (*S)-*product) and P450pra+T95F (selectivity 95% (*S)*-product) also a tiny fraction of *pro-R* NACs was observed, which is better in agreement with their strong stereoselectivities than the lack of *pro-R* NACs observed for these variants with the 5 × 22 ns MD simulations, which overestimates their selectivity. Also, P450pra and P450pra-T95F showed a wider range of conformations in 50 × 100 ps MD simulations, as indicated by plots of catalytic angle θ_2_ versus catalytic distance (Figure 5*versus*Figure 4). During the 50 × 100 ps simulations, these two variants also sample conformational space bordering and entering the range of *pro-R* NACs, while in the 5 long simulations they clearly sample less wide conformational space staying away from *pro-R* reactive conformations. Both these mutants are expected to reach into catalytically productive ranges since they do produce 6-epi-pravastatin, though as a minor product. Together, this suggests that the 50 × 100 ps MD simulations gave more complete conformational sampling than the 5 × 22 ns MD simulations, resulting in more accurate prediction at a lower computational cost.

## Discussion

In this study, we examined the use of computational methods to control the stereoselectivity of a cytochrome P450 hydroxylation reaction. In particular, we describe the application of the Rosetta CoupledMoves protocol^30^ to identify mutants for the asymmetric synthesis of epimerically pure pravastatin from compactin. The Rosetta enzyme design protocol generates a large number of design sequences and conformations which are usually ranked based on the Rosetta energy function.^26,34^ The CoupledMoves application includes backbone conformational changes in the design of such optimized enzyme variants. Incorporation of backbone motions is expected to improve the accuracy with which the enzyme–substrate complexes are modeled.

Previously, retrospective predictions obtained with CoupledMoves found good agreement with experimental results, both for ligand specificity and for binding affinity.^30^ Accordingly, we first demonstrated that mutations earlier found by directed evolution to switch the hydroxylation stereoselectivity from the formation of 6-epi-pravastatin (by wild-type CYP105AS1) to pravastatin (by P450pra) could be reproduced by CoupledMoves. These calculations revealed that the P450pra variant found by directed evolution indeed was present among a set of designs optimized by Rosetta for binding of compactin in a *pro-S* mode.

We subsequently used Rosetta CoupledMoves to support further improvement of the P450pra stereoselectivity. First, potentially influential positions for better compactin binding were detected by widening the Rosetta CoupledMoves search space to 14 positions. The two surface mutations (Q127R, A265N) were kept since they may influence the catalytic rate but are not expected to influence stereoselectivity.^66^ Among the Rosetta-optimized designs, only 8 positions appeared variable, and these were included in subsequent Rosetta calculations aimed at optimizing stereoselectivity. Mutations that were expected to promote hydroxylase epimeric selectivity toward pravastatin formation were detected by comparing the abundance of specific substitutions among a set of designs optimized for binding of compactin in a *pro-S* mode vs binding in a *pro-R* mode. Specific substitutions that exclusively occurred in *pro-S* virtual libraries were considered promising and their interactions with substrate were examined by visual inspection. These were tested experimentally in the P450pra background, and the two most promising substitutions were combined in P450pra to give a mutant (P450pra100) with near-perfect pravastatin: 6-epi-pravastatin product ratio (>99% pravastatin). The P450pra100 mutant also showed a higher affinity (lower *K*_d_) for compactin than the original variant (P450pra). Thus, the Rosetta CoupledMoves approach increased both affinity and stereoselectivity and can be used not only to explain the properties of known mutants^30^ but also to support the engineering of new improved variants.

Although the computation-aided approach reduced the variant pool to be tested in the laboratory to a very small set, we experienced the value of visual inspection during this process. Like in directed evolution, where library design is often steered by incorporating information from crystallographic, modeling, and bioinformatic studies,^95,96^ we used structural information to select positions for the Rosetta CoupledMoves search space and rational considerations to combine mutations in the final design. Such rational considerations are commonly used in the field of computational enzyme design.^92,97^ Following this approach, only eight mutants needed to be experimentally tested for finding a fully pravastatin-selective mutant, underlining the possibility of computational protocols to avoid the need for extensive screening by chiral chromatography.^17^ Although we do not show that Rosetta has the ability to design the P450pra100 variant in a single step starting with the wild-type CYP105AS1sequence, we did find that it is possible to stepwise reproduce all active-site mutations, including the ones previously found by directed evolution.

We also examined if the desired stereoselectivity could be predicted by molecular dynamics simulations. MD has previously been applied to P450s, *e.g.*, to understand the conformational dynamics and substrate binding mechanism,^43,57−61,98^ to evaluate the enhanced activity of experimentally characterized variants,^99^ to search for positions to mutate for altering substrate specificity,^32,45^ and to explain the specificity of mutants.^48−54,56^ Recently, MD runs of 22 ns were performed to examine the regioselectivity of steroid- and warfarin-hydroxylation, with scoring for near-attack conformations.^47,100^ However, due to the high computational cost, long MD simulations that are commonly used for studying protein dynamics cannot be applied for scoring large numbers of enzyme variants, and the user is bound to a restricted search space. A ranking method with high-throughput capacity is important since Rosetta and other computational design algorithms produce large numbers of primary designs with different sequences and conformations. Many of these have unrealistic substrate binding poses, as is also observed with docking simulations.^34,101,102^ The ranking of these designs is a critical step in the selection of variants that qualify for experimental verification if their number is to be low.^34^ QM approaches tend to give false-positive predictions on the catalytic prowess of designed enzymes as they lack the ability to sample alternative conformations. They do not well distinguish between good designs, in which the designed enzyme–substrate complex is feasible, and poor designs where unproductive binding modes are more realistic.^34,101^ MD simulations offer an attractive orthogonal tool for this ranking, decreasing the number of unstable substrate binding poses. The simulations cannot estimate the absolute barrier height but can predict the ability to form alternative catalytically competent conformations and thus can be used to predict stereoselectivity. This requires a computationally efficient protocol, yet good sampling of relevant conformations at the active site. For this purpose, multiple parallel ultrashort MD simulations have been studied.^15,33^

Multiple replicates with independent attribution of initial atom velocities offer a more rapid way of sampling than single simulations progressing over a longer timescale.^41,103−105^ In the case of P450s, loss of reactive conformations due to rapid substrate rotation in the active site has been described for small substrates.^106−109^ In the CASCO protocol for computational enzyme selectivity engineering, we used scoring for NAC frequencies along 5–20 MD trajectories of 10–100 ps each to rank enantioselective epoxide hydrolases and to eliminate variants with unreactive substrate binding orientations.^17,21^ Similarly, scoring of NACs along 40 × 10 ps MD trajectories was used to model the enantioselective conversion of 45 substrates with four different haloalkane dehalogenases,^21^ resulting in much better agreement with experiments than a single long MD simulation of 22 ns. These examples concern simulations with smaller molecules, which can reorient within the ps timescale.^21^ Such fast reorientation is possible because carbon atoms move at an average velocity of 2.5 Å/ps (at 298 K), making a simulation time of just a few picoseconds sufficient to reposition a small substrate.

To examine if short MD simulations can also be used for the bulky compound compactin, we explored both long (22 ns) and very short (100 ps) MD simulations of CYP105AS1 and derivatives thereof. In both cases, the results were in good agreement with the excellent epimeric excess found for the P450pra100 variant. Furthermore, the multiple parallel short MD simulations were found to reproduce with higher accuracy *pro-S* and *pro-R* enzyme selectivity for our P450 variants than the fewer long runs. This agrees with the established idea that at the start of an MD simulation, the protein will randomly relax into one of its many possible local energy minima after which jumps to another minimum occur rarely unless the simulations are run for a much longer time period.^9^ These alternative local minima can be accessed when using independent initialization of the MD trajectories by their initial atom velocities or other means.^104^

A limitation of the current protocol is some overestimation of the selectivity of some variants (Tables S6 and S9), both in the 5 × 22 ns MD simulations and in the 50 × 100 ps MD simulations. Accordingly, for P450wt no formation of its minor product (10% pravastatin) was detected. However, inspection of Figure 4 shows that (by 2 of the 3 criteria) NACs for *pro-S* are almost reached, suggesting the outcome is influenced by the strictness of NAC criteria. Alternatively, rare NACs may be undetected due to incomplete conformational sampling. With the mutants, the side-product NACs are more often observed in the 100 ps simulations that used more seeds and explored more diverse conformations. Possible ways to address under-sampling could be to use even more different trajectories, to use far longer MD simulations, or to use multiple orientations of the substrate as starting structures. However, such remedies are less attractive to include in a low-cost computational screening protocol.

Furthermore, the somewhat arbitrary nature of NAC criteria may play a role as well. NAC criteria are not optimized for methodological reasons; adapting the criteria by trial-and-error could easily lead to better agreement with the data due to over-fitting. Instead, NAC criteria are generated in a standard manner: distances between the reacting atoms closer than their Van der Waals contact distance and angles involving the reacting atoms within 20° from the corresponding angle in the transition state.^110^ This rigid nature of the NAC criteria is normally good enough to achieve satisfactory predictions of enantioselectivity^17,46,111^ but it is possible that wider criteria would more accurately distinguish reactive from nonreactive poses, which could help to diminish the overestimation of selectivity. The synergistic or additive effects causing the near-perfect selectivity of P450pra100 in comparison to the slightly lower selectivity of P450pra + V180M were not elucidated by MD simulations. Synergistic effects, even from remote mutations, have been explained by Acevedo-Rocha et al.^112^ through MD simulations, but the differences in experimental selectivity (97% vs >99%) between our P450pra mutants are much smaller than the effects of remote mutations studied in their work.

In conclusion, the results presented here show that the use of computational design can support the engineering of stereoselective P450 variants and indicate that including high-throughput short molecular dynamics simulations can improve the selection of preferred variants. This can support the replacement of time-consuming rational inspection steps in computational design protocols by more automated approaches.

## Supporting Information Available

Substrate and intermediate structures, experimental details, chromatograms, docking structures, description of libraries, and results of MD simulations (PDF)

## Notes

Dr. R.M. de Jong is affiliated with DSM, a global company active in Nutrition, Health & Bioscience.

## Abbreviations Used

MD: molecular dynamics
NAC: near-attack conformation

## References

[ref1] Mc Conathy J.; Owens M. J. Stereochemistry in Drug Action. Prim. Care Companion J. Clin. Psychiatry 2003, 05, 70–73. 10.4088/PCC.v05n0202. PMC353039 15156233

[ref2] Wang D.; Hu E. Structural Basis and Computational Modeling of Chiral Drugs. In Chiral Drugs, Lin G.-Q.; You Q.-D.; Cheng J.-F., Eds.; John Wiley & Sons, Inc: Hoboken, NJ, USA, 2011; pp 297–321.

[ref3] Nguyen L. A.; He H.; Pham-Huy C. Chiral Drugs: An Overview. Int. J. Biomed. Sci. 2006, 2, 85–100. 23674971 PMC3614593

[ref4] Caner H.; Groner E.; Levy L.; Agranat I. Trends in the Development of Chiral Drugs. Drug Discov. Today 2004, 9, 105–110. 10.1016/s1359-6446(03)02904-0. 15038394

[ref5] Zhang Z.-J.; Pan J.; Ma B.-D.; Xu J.-H. Efficient Biocatalytic Synthesis of Chiral Chemicals. Adv. Biochem. Eng. Biotechnol. 2016, 155, 55–106. 10.1007/10_2014_291. 25537446

[ref6] Li A.; Acevedo-Rocha C. G.; Sun Z.; Cox T.; Xu J. L.; Reetz M. T. Beating Bias in the Directed Evolution of Proteins: Combining High-Fidelity on-Chip Solid-Phase Gene Synthesis with Efficient Gene Assembly for Combinatorial Library Construction. ChemBioChem 2018, 19, 221–228. 10.1002/cbic.201700540. 29171900

[ref7] Arnold F. H. Directed Evolution: Bringing New Chemistry to Life. Angew. Chem., Int. Ed. 2018, 57, 4143–4148. 10.1002/anie.201708408. PMC5901037 29064156

[ref8] Zeymer C.; Hilvert D. Directed Evolution of Protein Catalysts. Annu. Rev. Biochem. 2018, 87, 131–157. 10.1146/annurev-biochem-062917-012034. 29494241

[ref9] Steiner K.; Schwab H. Recent Advances in Rational Approaches for Enzyme Engineering. Comput. Struct. Biotechnol. J. 2012, 2, e201209010 10.5936/csbj.201209010. 24688651 PMC3962183

[ref10] Röthlisberger D.; Khersonsky O.; Wollacott A. M.; Jiang L.; DeChancie J.; Betker J.; Gallaher J. L.; Althoff E. A.; Zanghellini A.; Dym O.; Albeck S.; Houk K. N.; Tawfik D. S.; Baker D. Kemp Elimination Catalysts by Computational Enzyme Design. Nature 2008, 453, 190–195. 10.1038/nature06879. 18354394

[ref11] Blomberg R.; Kries H.; Pinkas D. M.; Mittl P. R. E.; Grütter M. G.; Privett H. K.; Mayo S. L.; Hilvert D. Precision Is Essential for Efficient Catalysis in an Evolved Kemp Eliminase. Nature 2013, 503, 418–421. 10.1038/nature12623. 24132235

[ref12] Jiang L.; Althoff E. A.; Clemente F. R.; Doyle L.; Röthlisberger D.; Zanghellini A.; Gallaher J. L.; Betker J. L.; Tanaka F.; Barbas C. F.; Hilvert D.; Houk K. N.; Stoddard B. L.; Baker D. De Novo Computational Design of Retro-Aldol Enzymes. Science 2008, 319, 1387–1391. 10.1126/science.1152692. 18323453 PMC3431203

[ref13] Bolon D. N.; Mayo S. L. Enzyme-like Proteins by Computational Design. Proc. Natl. Acad. Sci. U.S.A. 2001, 98, 14274–14279. 10.1073/pnas.251555398. 11724958 PMC64672

[ref14] Korendovych I. V.; Kulp D. W.; Wu Y.; Cheng H.; Roder H.; DeGrado W. F. Design of a Switchable Eliminase. Proc. Natl. Acad. Sci. U. S. A. 2011, 108, 6823–6827. 10.1073/pnas.1018191108. 21482808 PMC3084051

[ref15] Wijma H. J.; Fürst M. J. L. J.; Janssen D. B. A Computational Library Design Protocol for Rapid Improvement of Protein Stability: FRESCO. Methods Mol. Biol. 2018, 1685, 69–85. 10.1007/978-1-4939-7366-8_5. 29086304

[ref16] Bednar D.; Beerens K.; Sebestova E.; Bendl J.; Khare S.; Chaloupkova R.; Prokop Z.; Brezovsky J.; Baker D.; Damborsky J. FireProt: Energy- and Evolution-Based Computational Design of Thermostable Multiple-Point Mutants. PLoS Comput. Biol. 2015, 11, e1004556 10.1371/journal.pcbi.1004556. 26529612 PMC4631455

[ref17] Wijma H. J.; Floor R. J.; Bjelic S.; Marrink S. J.; Baker D.; Janssen D. B. Enantioselective Enzymes by Computational Design and in Silico Screening. Angew. Chem. Int. Ed 2015, 54, 3726–3730. 10.1002/anie.201411415. 25651000

[ref18] Sumbalova L.; Stourac J.; Martinek T.; Bednar D.; Damborsky J. HotSpot Wizard 3.0: Web Server for Automated Design of Mutations and Smart Libraries Based on Sequence Input Information. Nucleic Acids Res. 2018, 46, W356–W362. 10.1093/nar/gky417. 29796670 PMC6030891

[ref19] Ferrario V.; Hansen N.; Pleiss J. Interpretation of Cytochrome P450 Monooxygenase Kinetics by Modeling of Thermodynamic Activity. J. Inorg. Biochem. 2018, 183, 172–178. 10.1016/j.jinorgbio.2018.02.016. 29530593

[ref20] Hediger M. R.; Jensen J. H.; De Vico L. BioFET-SIM Web Interface: Implementation and Two Applications. PLoS One 2012, 7, e45379 10.1371/journal.pone.0045379. 23056201 PMC3466287

[ref21] Wijma H. J.; Marrink S. J.; Janssen D. B. Computationally Efficient and Accurate Enantioselectivity Modeling by Clusters of Molecular Dynamics Simulations. J. Chem. Inf. Model. 2014, 54, 2079–2092. 10.1021/ci500126x. 24916632

[ref22] Mandell D. J.; Kortemme T. Backbone Flexibility in Computational Protein Design. Curr. Opin. Biotechnol. 2009, 20, 420–428. 10.1016/j.copbio.2009.07.006. 19709874

[ref23] Delgado J.; Radusky L. G.; Cianferoni D.; Serrano L. FoldX 5.0: Working with RNA, Small Molecules and a New Graphical Interface. Bioinformatics. 2019, 35, 4168–4169. 10.1093/bioinformatics/btz184. 30874800 PMC6792092

[ref24] Malisi C.; Schumann M.; Toussaint N. C.; Kageyama J.; Kohlbacher O.; Höcker B. Binding Pocket Optimization by Computational Protein Design. PLoS One 2012, 7, e52505 10.1371/journal.pone.0052505. 23300688 PMC3531388

[ref25] Privett H. K.; Kiss G.; Lee T. M.; Blomberg R.; Chica R. A.; Thomas L. M.; Hilvert D.; Houk K. N.; Mayo S. L. Iterative Approach to Computational Enzyme Design. Proc. Natl. Acad. Sci. U.S.A. 2012, 109, 3790–3795. 10.1073/pnas.1118082108. 22357762 PMC3309769

[ref26] Leaver-Fay A.; Tyka M.; Lewis S. M.; Lange O. F.; Thompson J.; Jacak R.; Kaufman K.; Renfrew P. D.; Smith C. A.; Sheffler W.; Davis I. W.; Cooper S.; Treuille A.; Mandell D. J.; Richter F.; Ban Y.-E. A.; Fleishman S. J.; Corn J. E.; Kim D. E.; Lyskov S.; Berrondo M.; Mentzer S.; Popović Z.; Havranek J. J.; Karanicolas J.; Das R.; Meiler J.; Kortemme T.; Gray J. J.; Kuhlman B.; Baker D.; Bradley P. ROSETTA3: An Object-Oriented Software Suite for the Simulation and Design of Macromolecules. Methods Enzymol. 2011, 487, 545–574. 10.1016/B978-0-12-381270-4.00019-6. 21187238 PMC4083816

[ref27] Richter F.; Leaver-Fay A.; Khare S. D.; Bjelic S.; Baker D. De Novo Enzyme Design Using Rosetta3. PLoS One 2011, 6, e19230 10.1371/journal.pone.0019230. 21603656 PMC3095599

[ref28] Davis I. W.; Arendall W. B.; Richardson D. C.; Richardson J. S. The Backrub Motion: How Protein Backbone Shrugs When a Sidechain Dances. Structure 2006, 14, 265–274. 10.1016/j.str.2005.10.007. 16472746

[ref29] Friedland G. D.; Linares A. J.; Smith C. A.; Kortemme T. A Simple Model of Backbone Flexibility Improves Modeling of Side-Chain Conformational Variability. J. Mol. Biol. 2008, 380, 757–774. 10.1016/j.jmb.2008.05.006. 18547586 PMC3574579

[ref30] Ollikainen N.; de Jong R. M.; Kortemme T. Coupling Protein Side-Chain and Backbone Flexibility Improves the Re-Design of Protein-Ligand Specificity. PLoS Comput. Biol. 2015, 11, e1004335 10.1371/journal.pcbi.1004335. 26397464 PMC4580623

[ref31] Loshbaugh A. L.; Kortemme T. Comparison of Rosetta Flexible-Backbone Computational Protein Design Methods on Binding Interactions. Proteins 2020, 88, 206–226. 10.1002/prot.25790. 31344278 PMC6901717

[ref32] Dodani S. C.; Kiss G.; Cahn J. K. B.; Su Y.; Pande V. S.; Arnold F. H. Discovery of a Regioselectivity Switch in Nitrating P450s Guided by Molecular Dynamics Simulations and Markov Models. Nat. Chem. 2016, 8, 419–425. 10.1038/nchem.2474. 27102675 PMC4843824

[ref33] Meng Q.; Capra N.; Palacio C. M.; Lanfranchi E.; Otzen M.; van Schie L. Z.; Rozeboom H. J.; Thunnissen A.-M. W. H.; Wijma H. J.; Janssen D. B. Robust ω-Transaminases by Computational Stabilization of the Subunit Interface. ACS Catal. 2020, 10, 2915–2928. 10.1021/acscatal.9b05223. 32953233 PMC7493286

[ref34] Kiss G.; Röthlisberger D.; Baker D.; Houk K. N. Evaluation and Ranking of Enzyme Designs. Protein Sci. 2010, 19, 1760–1773. 10.1002/pro.462. 20665693 PMC2975139

[ref35] Pratter S. M.; Konstantinovics C.; Di Giuro C. M. L.; Leitner E.; Kumar D.; de Visser S. P.; Grogan G.; Straganz G. D. Inversion of Enantioselectivity of a Mononuclear Non-Heme Iron(II)-Dependent Hydroxylase by Tuning the Interplay of Metal-Center Geometry and Protein Structure. Angew. Chem., Int. Ed. 2013, 52, 9677–9681. 10.1002/anie.201304633. 23881738

[ref36] Hur S.; Bruice T. C. The near Attack Conformation Approach to the Study of the Chorismate to Prephenate Reaction. Proc. Natl. Acad. Sci. U.S.A. 2003, 100, 12015–12020. 10.1073/pnas.1534873100. 14523243 PMC218705

[ref37] Arabnejad H.; Bombino E.; Colpa D. I.; Jekel P. A.; Trajkovic M.; Wijma H. J.; Janssen D. B. Computational Design of Enantiocomplementary Epoxide Hydrolases for Asymmetric Synthesis of Aliphatic and Aromatic Diols. ChemBioChem 2020, 21, 1893–1904. 10.1002/cbic.201900726. 31961471 PMC7383614

[ref38] Li R.; Wijma H. J.; Song L.; Cui Y.; Otzen M.; Tian Y.; Du J.; Li T.; Niu D.; Chen Y.; Feng J.; Han J.; Chen H.; Tao Y.; Janssen D. B.; Wu B. Computational Redesign of Enzymes for Regio- and Enantioselective Hydroamination. Nat. Chem. Biol. 2018, 14, 664–670. 10.1038/s41589-018-0053-0. 29785057

[ref39] Bruice T. C. A View at the Millennium: The Efficiency of Enzymatic Catalysis. Acc. Chem. Res. 2002, 35, 139–148. 10.1021/ar0001665. 11900517

[ref40] Warshel A.; Sharma P. K.; Kato M.; Xiang Y.; Liu H.; Olsson M. H. M. Electrostatic Basis for Enzyme Catalysis. Chem. Rev. 2006, 106, 3210–3235. 10.1021/cr0503106. 16895325

[ref41] Caves L. S. D.; Evanseck J. D.; Karplus M. Locally Accessible Conformations of Proteins: Multiple Molecular Dynamics Simulations of Crambin. Protein Sci. 1998, 7, 649–666. 10.1002/pro.5560070314. 9541397 PMC2143962

[ref42] Ekroos M.; Sjogren T. Structural Basis for Ligand Promiscuity in Cytochrome P450 3A4. Proc. Natl. Acad. Sci. U.S.A. 2006, 103, 13682–13687. 10.1073/pnas.0603236103. 16954191 PMC1564212

[ref43] Caddell Haatveit K.; Garcia-Borràs M.; Houk K. N. Computational Protocol to Understand P450 Mechanisms and Design of Efficient and Selective Biocatalysts. Front. Chem. 2019, 6, 663 10.3389/fchem.2018.00663. 30687699 PMC6336901

[ref44] Li Z.; Burnell D. J.; Boyd R. J. Computational Study of Engineered Cytochrome P450-Catalyzed C–H Amination: The Origin of the Regio- and Stereoselectivity. J. Phys. Chem. B 2017, 121, 10859–10868. 10.1021/acs.jpcb.7b10256. 29131622

[ref45] Yang S.; DeMars M. D.; Grandner J. M.; Olson N. M.; Anzai Y.; Sherman D. H.; Houk K. N. Computational-Based Mechanistic Study and Engineering of Cytochrome P450 MycG for Selective Oxidation of 16-Membered Macrolide Antibiotics. J. Am. Chem. Soc. 2020, 142, 17981–17988. 10.1021/jacs.0c04388. 33030347 PMC7720209

[ref46] Bracco P.; Wijma H. J.; Nicolai B.; Buitrago J. A. R.; Klünemann T.; Vila A.; Schrepfer P.; Blankenfeldt W.; Janssen D. B.; Schallmey A. CYP154C5 Regioselectivity in Steroid Hydroxylation Explored by Substrate Modifications and Protein Engineering. Chembiochem 2021, 22, 1099–1110. 10.1002/cbic.202000735. 33145893 PMC8048783

[ref47] Seifert A.; Tatzel S.; Schmid R. D.; Pleiss J. Multiple Molecular Dynamics Simulations of Human P450 Monooxygenase CYP2C9: The Molecular Basis of Substrate Binding and Regioselectivity toward Warfarin. Proteins 2006, 64, 147–155. 10.1002/prot.20951. 16639745

[ref48] Venkataraman H.; Beer S. B. A.; de Bergen L. A. H.; van Essen N.; van Geerke D. P.; Vermeulen N. P. E.; Commandeur J. N. M. A Single Active Site Mutation Inverts Stereoselectivity of 16-Hydroxylation of Testosterone Catalyzed by Engineered Cytochrome P450 BM3. Chembiochem 2012, 13, 520–523. 10.1002/cbic.201100750. 22275147

[ref49] Seifert A.; Vomund S.; Grohmann K.; Kriening S.; Urlacher V. B.; Laschat S.; Pleiss J. Rational Design of a Minimal and Highly Enriched CYP102A1 Mutant Library with Improved Regio-, Stereo- and Chemoselectivity. ChemBioChem 2009, 10, 853–861. 10.1002/cbic.200800799. 19222039

[ref50] Luirink R. A.; Verkade-Vreeker M. C. A.; Commandeur J. N. M.; Geerke D. P. A Modified Arrhenius Approach to Thermodynamically Study Regioselectivity in Cytochrome P450-Catalyzed Substrate Conversion. ChemBioChem 2020, 21, 1461–1472. 10.1002/cbic.201900751. 31919943 PMC7318578

[ref51] Capoferri L.; Leth R.; ter Haar E.; Mohanty A. K.; Grootenhuis P. D. J.; Vottero E.; Commandeur J. N. M.; Vermeulen N. P. E.; Jørgensen F. S.; Olsen L.; Geerke D. P. Insights into Regioselective Metabolism of Mefenamic Acid by Cytochrome P450 BM3 Mutants through Crystallography, Docking, Molecular Dynamics, and Free Energy Calculations. Proteins 2016, 84, 383–396. 10.1002/prot.24985. 26757175

[ref52] Seifert A.; Antonovici M.; Hauer B.; Pleiss J. An Efficient Route to Selective Bio-Oxidation Catalysts: An Iterative Approach Comprising Modeling, Diversification, and Screening, Based on CYP102A1. ChemBioChem 2011, 12, 1346–1351. 10.1002/cbic.201100067. 21591046

[ref53] Weber E.; Seifert A.; Antonovici M.; Geinitz C.; Pleiss J.; Urlacher V. B. Screening of a Minimal Enriched P450 BM3 Mutant Library for Hydroxylation of Cyclic and Acyclic Alkanes. Chem. Commun. 2011, 47, 944–946. 10.1039/c0cc02924f. 21079837

[ref54] Eichler A.; Gricman Ł.; Herter S.; Kelly P. P.; Turner N. J.; Pleiss J.; Flitsch S. L. Enantioselective Benzylic Hydroxylation Catalysed by P450 Monooxygenases: Characterisation of a P450cam Mutant Library and Molecular Modelling. ChemBioChem 2016, 17, 426–432. 10.1002/cbic.201500536. 26698167

[ref55] Paulsen M. D.; Manchester J. I.; Ornstein R. L. Using Molecular Modeling and Molecular Dynamics Simulation to Predict P450 Oxidation Products. Methods Enzymol. 1996, 272, 347–357. 10.1016/S0076-6879(96)72040-2. 8791794

[ref56] de Graaf C.; Oostenbrink C.; J Keizers P.; A van Vugt-Lussenburg B.; B van Waterschoot R.; Tschirret-Guth R.; M Commandeur J.; E Vermeulen N. Molecular Modeling-Guided Site-Directed Mutagenesis of Cytochrome P450 2D6. Curr. Drug Metab. 2007, 8, 59–77. 10.2174/138920007779315062. 17266524

[ref57] Bren U.; Fuchs J. E.; Oostenbrink C. Cooperative Binding of Aflatoxin B _1_ by Cytochrome P450 3A4: A Computational Study. Chem. Res. Toxicol. 2014, 27, 2136–2147. 10.1021/tx5004062. 25398138

[ref58] Jandova Z.; Gill S. C.; Lim N. M.; Mobley D. L.; Oostenbrink C. Binding Modes and Metabolism of Caffeine. Chem. Res. Toxicol. 2019, 32, 1374–1383. 10.1021/acs.chemrestox.9b00030. 31132250 PMC6635882

[ref59] Santos R.; Hritz J.; Oostenbrink C. Role of Water in Molecular Docking Simulations of Cytochrome P450 2D6. J. Chem. Inf. Model. 2010, 50, 146–154. 10.1021/ci900293e. 19899781

[ref60] Rifai E. A.; Ferrario V.; Pleiss J.; Geerke D. P. Combined Linear Interaction Energy and Alchemical Solvation Free-Energy Approach for Protein-Binding Affinity Computation. J. Chem. Theory Comput. 2020, 16, 1300–1310. 10.1021/acs.jctc.9b00890. 31894691 PMC7017367

[ref61] Stjernschantz E.; van Vugt-Lussenburg B. M. A.; Bonifacio A.; de Beer S. B. A.; van der Zwan G.; Gooijer C.; Commandeur J. N. M.; Vermeulen N. P. E.; Oostenbrink C. Structural Rationalization of Novel Drug Metabolizing Mutants of Cytochrome P450 BM3. Proteins 2008, 71, 336–352. 10.1002/prot.21697. 17957765

[ref62] Stancu C.; Sima A. Statins: Mechanism of Action and Effects. J. Cell. Mol. Med. 2001, 5, 378–387. 10.1111/j.1582-4934.2001.tb00172.x. 12067471 PMC6740083

[ref63] Bener A.; Dogan M.; Barakat L.; Al-Hamaq A. O. A. A. Comparison of Efficacy, Safety, and Cost-Effectiveness of Various Statins in Dyslipidemic Diabetic Patients. Indian J. Pharmacol. 2014, 46, 88 10.4103/0253-7613.125184. 24550591 PMC3912814

[ref64] Willrich M. A. V.; Hirata M. H.; Hirata R. D. C. Statin Regulation of CYP3A4 and CYP3A5 Expression. Pharmacogenomics 2009, 10, 1017–1024. 10.2217/pgs.09.42. 19530969

[ref65] Neuvonen P. J. Drug Interactions with HMG-CoA Reductase Inhibitors (Statins): The Importance of CYP Enzymes, Transporters and Pharmacogenetics. Curr. Opin. Investig. Drugs Lond. Engl. 2000 2010, 11, 323–332. 20178046

[ref66] McLean K. J.; Hans M.; Meijrink B.; van Scheppingen W. B.; Vollebregt A.; Tee K. L.; van der Laan J.-M.; Leys D.; Munro A. W.; van den Berg M. A. Single-Step Fermentative Production of the Cholesterol-Lowering Drug Pravastatin via Reprogramming of *Penicillium chrysogenum*. Proc. Natl. Acad. Sci. U.S.A. 2015, 112, 2847–2852. 10.1073/pnas.1419028112. 25691737 PMC4352836

[ref67] Watts K. S.; Dalal P.; Murphy R. B.; Sherman W.; Friesner R. A.; Shelley J. C. ConfGen: A Conformational Search Method for Efficient Generation of Bioactive Conformers. J. Chem. Inf. Model. 2010, 50, 534–546. 10.1021/ci100015j. 20373803

[ref68] Madhavi Sastry G.; Adzhigirey M.; Day T.; Annabhimoju R.; Sherman W. Protein and Ligand Preparation: Parameters, Protocols, and Influence on Virtual Screening Enrichments. J. Comput. Aided Mol. Des. 2013, 27, 221–234. 10.1007/s10822-013-9644-8. 23579614

[ref69] Harder E.; Damm W.; Maple J.; Wu C.; Reboul M.; Xiang J. Y.; Wang L.; Lupyan D.; Dahlgren M. K.; Knight J. L.; Kaus J. W.; Cerutti D. S.; Krilov G.; Jorgensen W. L.; Abel R.; Friesner R. A. OPLS3: A Force Field Providing Broad Coverage of Drug-like Small Molecules and Proteins. J. Chem. Theory Comput. 2016, 12, 281–296. 10.1021/acs.jctc.5b00864. 26584231

[ref70] Kuhn B.; Jacobsen W.; Christians U.; Benet L. Z.; Kollman P. A. Metabolism of Sirolimus and Its Derivative Everolimus by Cytochrome P450 3A4: Insights from Docking, Molecular Dynamics, and Quantum Chemical Calculations. J. Med. Chem. 2001, 44, 2027–2034. 10.1021/jm010079y. 11384247

[ref71] Oláh J.; Mulholland A. J.; Harvey J. N. Understanding the Determinants of Selectivity in Drug Metabolism through Modeling of Dextromethorphan Oxidation by Cytochrome P450. Proc. Natl. Acad. Sci. U.S.A. 2011, 108, 6050–6055. 10.1073/pnas.1010194108. 21444768 PMC3076858

[ref72] Rittle J.; Green M. T. Cytochrome P450 Compound I: Capture, Characterization, and C-H Bond Activation Kinetics. Science 2010, 330, 933–937. 10.1126/science.1193478. 21071661

[ref73] Poulos T. L. Cytochrome P450 Flexibility. Proc. Natl. Acad. Sci. U.S.A. 2003, 100, 13121–13122. 10.1073/pnas.2336095100. 14597705 PMC263725

[ref74] The PyMOL Molecular Graphics System, Version 2.0, Schrödinger, LLC: Https://Pymol.Org/2/.

[ref75] Trott O.; Olson A. J. AutoDock Vina: Improving the Speed and Accuracy of Docking with a New Scoring Function, Efficient Optimization and Multithreading. J. Comput. Chem. 2010, 31, 455–461. 10.1002/jcc.21334. 19499576 PMC3041641

[ref76] Krieger E.; Vriend G. YASARA View - Molecular Graphics for All Devices - from Smartphones to Workstations. Bioinformatics 2014, 30, 2981–2982. 10.1093/bioinformatics/btu426. 24996895 PMC4184264

[ref77] Suzek B. E.; Huang H.; McGarvey P.; Mazumder R.; Wu C. H. UniRef: Comprehensive and Non-Redundant UniProt Reference Clusters. Bioinformatics 2007, 23, 1282–1288. 10.1093/bioinformatics/btm098. 17379688

[ref78] Altschul S. F.; Gish W.; Miller W.; Myers E. W.; Lipman D. J. Basic Local Alignment Search Tool. J. Mol. Biol. 1990, 215, 403–410. 10.1016/S0022-2836(05)80360-2. 2231712

[ref79] Hall T. A.; H T. A. BioEdit: A User-Friendly Biological Sequence Alignment Editor and Analysis Program for Windows 95/98/NT, Nucleic Acids Symp. Ser., pp 95–98.

[ref80] Morris G. M.; Huey R.; Lindstrom W.; Sanner M. F.; Belew R. K.; Goodsell D. S.; Olson A. J. AutoDock4 and AutoDockTools4: Automated Docking with Selective Receptor Flexibility. J. Comput. Chem. 2009, 30, 2785–2791. 10.1002/jcc.21256. 19399780 PMC2760638

[ref81] Krieger E.; Darden T.; Nabuurs S. B.; Finkelstein A.; Vriend G. Making Optimal Use of Empirical Energy Functions: Force-Field Parameterization in Crystal Space. Proteins 2004, 57, 678–683. 10.1002/prot.20251. 15390263

[ref82] Jakalian A.; Jack D. B.; Bayly C. I. Fast, Efficient Generation of High-Quality Atomic Charges. AM1-BCC Model: II. Parameterization and Validation. J. Comput. Chem. 2002, 23, 1623–1641. 10.1002/jcc.10128. 12395429

[ref83] Shahrokh K.; Orendt A.; Yost G. S.; Cheatham T. E. Quantum Mechanically Derived AMBER-Compatible Heme Parameters for Various States of the Cytochrome P450 Catalytic Cycle. J. Comput. Chem. 2012, 33, 119–133. 10.1002/jcc.21922. 21997754 PMC3242737

[ref84] Shaik S.; Lai W.; Chen H.; Wang Y. The Valence Bond Way: Reactivity Patterns of Cytochrome P450 Enzymes and Synthetic Analogs. Acc. Chem. Res. 2010, 43, 1154–1165. 10.1021/ar100038u. 20527755

[ref85] Blanksby S. J.; Ellison G. B. Bond Dissociation Energies of Organic Molecules. Acc. Chem. Res. 2003, 36, 255–263. 10.1021/ar020230d. 12693923

[ref86] Xiu-Juan Q.; Yong F.; Lei L.; Qing-Xiang G. Assessment of Performance of G3B3 and CBS-QB3 Methods in Calculation of Bond Dissociation Energies. Chin. J. Chem. 2005, 23, 194–199. 10.1002/cjoc.200590194.

[ref87] McIver L.; Leadbeater C.; Campopiano D. J.; Baxter R. L.; Daff S. N.; Chapman S. K.; Munro A. W. Characterisation of Flavodoxin NADP+ Oxidoreductase and Flavodoxin; Key Components of Electron Transfer in *Escherichia coli*. Eur. J. Biochem. 1998, 257, 577–585. 10.1046/j.1432-1327.1998.2570577.x. 9839946

[ref88] Leadbeater C.; Mciver L.; Campopiano D. J.; Webster S. P.; Baxter R. L.; Kelly S. M.; Price N. C.; Lysek D. A.; Noble M. A.; Chapman S. K.; Munro A. W. Probing the NADPH-Binding Site of *Escherichia coli* Flavodoxin Oxidoreductase. Biochem. J. 2000, 352, 257–266. 10.1042/bj3520257. 11085917 PMC1221455

[ref89] Berry E. A.; Trumpower B. L. Simultaneous Determination of Hemes a, b, and c from Pyridine Hemochrome Spectra. Anal. Biochem. 1987, 161, 1–15. 10.1016/0003-2697(87)90643-9. 3578775

[ref90] McLean K. J.; Marshall K. R.; Richmond A.; Hunter I. S.; Fowler K.; Kieser T.; Gurcha S. S.; Besra G. S.; Munro A. W. Azole Antifungals Are Potent Inhibitors of Cytochrome P450 Mono-Oxygenases and Bacterial Growth in Mycobacteria and Streptomycetes. Microbiol. Read. Engl. 2002, 148, 2937–2949. 10.1099/00221287-148-10-2937. 12368427

[ref91] Morrison J. F. Kinetics of the Reversible Inhibition of Enzyme-Catalysed Reactions by Tight-Binding Inhibitors. Biochim. Biophys. Acta 1969, 185, 269–286. 10.1016/0005-2744(69)90420-3. 4980133

[ref92] Nivón L. G.; Bjelic S.; King C.; Baker D. Automating Human Intuition for Protein Design. Proteins Struct. Funct. Bioinforma. 2014, 82, 858–866. 10.1002/prot.24463. 24265170

[ref93] Genheden S.; Ryde U. Will Molecular Dynamics Simulations of Proteins Ever Reach Equilibrium?. Phys. Chem. Chem. Phys. 2012, 14, 8662 10.1039/c2cp23961b. 22614001

[ref94] Gasteiger J. Empirical Methods for the Calculation of Physicochemical Data of Organic Compounds. In Physical Property Prediction in Organic Chemistry, Jochum C.; Hicks M. G.; Sunkel J., Eds.; Springer Berlin Heidelberg: Berlin, Heidelberg, 1988; pp 119–138.

[ref95] Sun Z.; Wikmark Y.; Bäckvall J.-E.; Reetz M. T. New Concepts for Increasing the Efficiency in Directed Evolution of Stereoselective Enzymes. Chem. Weinh. Bergstr. Ger. 2016, 22, 5046–5054. 10.1002/chem.201504406. 26914401

[ref96] Yang K. K.; Wu Z.; Arnold F. H. Machine-Learning-Guided Directed Evolution for Protein Engineering. Nat. Methods 2019, 16, 687–694. 10.1038/s41592-019-0496-6. 31308553

[ref97] Ulge U. Y.; Baker D. A.; Monnat R. J. Comprehensive Computational Design of MCreI Homing Endonuclease Cleavage Specificity for Genome Engineering. Nucleic Acids Res. 2011, 39, 4330–4339. 10.1093/nar/gkr022. 21288879 PMC3105429

[ref98] Stjernschantz E.; Vermeulen N. P.; Oostenbrink C. Computational Prediction of Drug Binding and Rationalisation of Selectivity towards Cytochromes P450. Expert Opin. Drug Metab. Toxicol. 2008, 4, 513–527. 10.1517/17425255.4.5.513. 18484912

[ref99] Narayan A. R. H.; Jiménez-Osés G.; Liu P.; Negretti S.; Zhao W.; Gilbert M. M.; Ramabhadran R. O.; Yang Y.-F.; Furan L. R.; Li Z.; Podust L. M.; Montgomery J.; Houk K. N.; Sherman D. H. Enzymatic Hydroxylation of an Unactivated Methylene C–H Bond Guided by Molecular Dynamics Simulations. Nat. Chem. 2015, 7, 653–660. 10.1038/nchem.2285. 26201742 PMC4518477

[ref100] Jóźwik I. K.; Kiss F. M.; Gricman Ł.; Abdulmughni A.; Brill E.; Zapp J.; Pleiss J.; Bernhardt R.; Thunnissen A.-M. W. H. Structural Basis of Steroid Binding and Oxidation by the Cytochrome P450 CYP109E1 from *Bacillus megaterium*.. FEBS J. 2016, 283, 4128–4148. 10.1111/febs.13911. 27686671 PMC5132081

[ref101] Mladenovic M.; Ansorg K.; Fink R. F.; Thiel W.; Schirmeister T.; Engels B. Atomistic Insights into the Inhibition of Cysteine Proteases: First QM/MM Calculations Clarifying the Stereoselectivity of Epoxide-Based Inhibitors. J. Phys. Chem. B 2008, 112, 11798–11808. 10.1021/jp803895f. 18712902

[ref102] Wijma H. J. In Understanding Enzymes: Function, Design, Engineering, and Analysis, Svendsen A., Ed.; Pan Stanford Publishing Pte. Ltd.: Singapore, 2016; pp 805–833, 24 p.

[ref103] Genheden S.; Ryde U. How to Obtain Statistically Converged MM/GBSA Results. J. Comput. Chem. 2010, 31, 837–846. 10.1002/jcc.21366. 19598265

[ref104] Genheden S.; Ryde U. A Comparison of Different Initialization Protocols to Obtain Statistically Independent Molecular Dynamics Simulations. J. Comput. Chem. 2011, 32, 187–195. 10.1002/jcc.21546. 21132839

[ref105] Elofsson A.; Nilsson L. How Consistent Are Molecular Dynamics Simulations? Comparing Structure and Dynamics in Reduced and Oxidized *Escherichia coli* Thioredoxin. J. Mol. Biol. 1993, 233, 766–780. 10.1006/jmbi.1993.1551. 8411178

[ref106] Paulsen M. D.; Ornstein R. L. Substrate Mobility in Thiocamphor-Bound Cytochrome P450_cam_: An Explanation of the Conflict between the Observed Product Profile and the X-Ray Structure. Protein Eng. Des. Sel. 1993, 6, 359–365. 10.1093/protein/6.4.359. 8332592

[ref107] Loida P. J.; Sligar S. G.; Paulsen M. D.; Arnold G. E.; Ornstein R. L. Stereoselective Hydroxylation of Norcamphor by Cytochrome P450cam. J. Biol. Chem. 1995, 270, 5326–5330. 10.1074/jbc.270.10.5326. 7890644

[ref108] Manchester J. I.; Ornstein R. L. Enzyme-Catalyzed Dehalogenation of Pentachloroethane: Why F87W-Cytochrome P450cam Is Faster than Wild Type. Protein Eng. Des. Sel. 1995, 8, 801–807. 10.1093/protein/8.8.801. 8637849

[ref109] Filipovic D.; Paulsen M. D.; Loida P. J.; Sligar S. G.; Ornstein R. L. Ethylbenzene Hydroxylation by Cytochrome P450cam. Biochem. Biophys. Res. Commun. 1992, 189, 488–495. 10.1016/0006-291X(92)91584-D. 1449498

[ref110] Bruice T. C. Computational Approaches: Reaction Trajectories, Structures, and Atomic Motions. Enzyme Reactions and Proficiency. Chem. Rev. 2006, 106, 3119–3139. 10.1021/cr050283j. 16895321

[ref111] Meng Q.; Ramírez-Palacios C.; Capra N.; Hooghwinkel M. E.; Thallmair S.; Rozeboom H. J.; Thunnissen A.-M. W. H.; Wijma H. J.; Marrink S. J.; Janssen D. B. Computational Redesign of an ω-Transaminase from *Pseudomonas jessenii* for Asymmetric Synthesis of Enantiopure Bulky Amines. ACS Catal. 2021, 11, 10733–10747. 10.1021/acscatal.1c02053. 34504735 PMC8419838

[ref112] Acevedo-Rocha C. G.; Li A.; D’Amore L.; Hoebenreich S.; Sanchis J.; Lubrano P.; Ferla M. P.; Garcia-Borràs M.; Osuna S.; Reetz M. T. Pervasive Cooperative Mutational Effects on Multiple Catalytic Enzyme Traits Emerge via Long-Range Conformational Dynamics. Nat. Commun. 2021, 12, 1621 10.1038/s41467-021-21833-w. 33712579 PMC7955134

