# Supplementary material for: Computation-Aided Engineering of Cytochrome P450 for the Production of Pravastatin
Source: ACS Catal. 2022 Nov 28;12(24):15028–44. doi: 10.1021/acscatal.2c03974 (PMC9764288; doi:10.1021/acscatal.2c03974)
Supplement: Supplementary file 1 — cs2c03974_si_001.pdf [file cs2c03974_si_001.pdf]

Supplementary Information

Computation-aided engineering of cytochrome P450 for production of pravastatin

Mark A. Ashworth,[1§] Elvira Bombino,[2§] René M. de Jong,[3] Hein J. Wijma,[2] Dick B. Janssen,[2,*] Kirsty J. McLean[1,4] and Andrew W. Munro[1]

[1] Manchester Institute of Biotechnology, School of Chemistry, the University of Manchester, Manchester M1 7DN, United Kingdom.

[2] Department of Biochemistry, Groningen Biomolecular Sciences and Biotechnology Institute, University of Groningen, Nijenborgh 4, Groningen, Netherlands.

[3] DSM Food & Beverage, Alexander Fleminglaan 1, 2613 AX, Delft, the Netherlands.

[4] Department of Biological and Geographical Sciences, School of Applied Sciences, University of Huddersfield, Huddersfield HD1 3DH

[§] These authors contributed equally to this work

[*] Corresponding author. E-mail: d.b.janssen@rug.nl.

`

Supplementary Tables

Table S1. Distribution of amino acids at 3 target positions among CYP105AS1-derived designs optimized for *pro-S* compactin by Rosetta Coupled Moves. The three target positions were mutated in the directed evolution variant P450pra. Data refer to the 1,748 low-energy solutions found by Rosetta.

**Template**	**Frequency of occurrence (%)**																			
**(CYP105AS1)**	**A**	**R**	**N**	**D**	**C**	**E**	**Q**	**G**	**H**	**I**	**L**	**K**	**M**	**F**	**P**	**S**	**T**	**W**	**Y**	**V**
**I95**	14.7	0.00	1.59	0.00	2.39	0.40	1.20	5.98	3.19	6.37	0.40	0.00	0.80	20.72	0.00	4.78	4.78	14.7	9.16	8.76
**A180**	27.9	0.00	0.00	0.00	3.98	0.00	0.00	14.7	0.00	5.58	0.00	0.00	8.37	0.00	0.00	14.7	17.9	0.00	0.00	6.77
**L236**	9.16	0.00	3.98	0.00	5.18	1.59	9.96	0.00	0.00	27.9	22.3	0.00	0.80	0.00	0.00	1.20	7.57	0.00	0.00	10.4

Table S2. Sequence and Rosetta energy units for the 10 sequences most frequently predicted by Rosetta CoupledMoves when varying the 3 positions mutated by directed evolution in variant P450pra. Amino acids I95, A180 and L236 refer to the CYP105AS1 template.

**Ranking**	**I95**	**A180**	**L236**	**Rosetta energy**
1	V	V	I	133,09
2	W	T	I	133,82
3	F	V	I	133,84
4	V	T	I	134,07
5	Y	V	I	134,10
6	A	V	I	134,97
7	I	V	I	135,01
8 (P450pra)	T	V	I	135,06
9	H	V	I	135,58
10	F	T	I	135,74

Table S3. Distribution of amino acids at 14 target positions optimized for compactin binding by Rosetta Coupled_Moves. Data refer to the initial 12,569 low-energy designs generated by Rosetta using a *pro-S* starting conformation. The set was subsequently reduced to 9,052 unique sequences by removing high-energy duplicates.

**Template (P450pra) posn**	**Frequency of occurrence (%)**																			
	**A**	**R**	**N**	**D**	**C**	**E**	**Q**	**G**	**H**	**I**	**L**	**K**	**M**	**F**	**P**	**S**	**T**	**W**	**Y**	**V**
**F76**	0.71	0.75	0.29	0	0.75	0.08	0.99	0.02	0.91	7.17	1.38	0.35	0.56	45.5	0	0.2	3.82	4.08	0.23	32.2
**P80**	54.3	0	0.08	0	2.08	0	0	4.24	2.59	0	0.25	0	0	29.4	0.96	2.85	2.76	0	0.44	0
**W93**	0	0	0	0	0	0	0	0	0.06	0	0	0	0	0.15	0	0	0	99.8	0	0
**T95**	4.67	0	1.68	0	0.46	0	0	0.19	0.2	0.64	0	0	0	6.64	0	1.51	5.81	18.9	0.39	58.9
**M179**	38.2	0	0.07	0.12	0.97	0.03	1.23	4.99	0	0.03	2.55	0	51.3	0	0	0.13	0.32	0	0	0
**V180**	2.22	0	0	0	0.2	0	0.03	0.07	0.36	0.57	0	0	3.18	0	0	0.12	16.3	0	0	77.0
**V182**	4.18	0.1	0.8	0.6	4.57	0.04	0.08	1.01	3.62	1.1	23.4	0	5.16	0.49	0	1.66	24.7	0.04	3.92	45.4
**L235**	57.1	0	0.42	0.31	3.06	0.02	0	0.95	0.24	0.1	8.13	0	0	0	0	2.45	26.9	0	0	0.17
**I236**	0.38	0	0	0	0	0	0.02	0	0	99.1	0.24	0	0	0	0	0	0.07	0	0	0.23
**V239**	8.72	0	0	0.04	0.82	0	0	0	0	72.6	0	0	0	0	0	0.04	1.33	0	0	16.4
**V282**	0.02	0	0	0	0	0	0.01	0	0.22	64.7	4.93	0	1.29	3.98	0	0	0	0	0	24.8
**T286**	0.39	0.55	6.22	0	0.97	0	0.02	0.09	4.43	37.6	3.84	0.21	3.04	21.0	0	0	1.43	1.98	2.11	15.9
**A388**	64.2	0	0.03	0.04	0.12	0	0.32	27.6	0.09	0	3.04	0	4.33	0	0	0.19	0.06	0	0	0
**A389**	76.6	0	0	0	0.27	0	0	0.43	0	0	0	0	0	0	0	22.65	0.09	0	0	0

`

Table S4. Frequencies of amino acids at variable positions among Rosetta CoupledMoves designs of P450pra optimized for compactin binding in a *pro-S* compactin binding mode. Amino acids occurring in this set but not among designs optimized for binding compactin in a *pro-R* mode (Table S5) are labeled in red.

Position	1st	(%)	2nd	(%)	3rd	(%)	4th	(%)	5th	(%)
F76	F	32.2	V	31.0	I	9.16	T	5.71	W	4.77
P80	A	67.3	F	20.7	P	4.04	G	3.42	S	2.78
T95	V	61.0	A	8.19	F	7.70	W	7.14	T	7.14
M179	M	69.8	A	17.7	G	6.76	L	4.62	Q	0.32
V180	V	67.0	T	24.7	A	3.28	I	2.31	M	1.05
V182	V	34.2	T	22.0	A	8.93	C	6.29	M	5.68
T286	F	31.7	V	22.4	N	11.2	I	9.92	H	8.52
A388	A	58.9	G	24.9	M	7.84	F	3.63	Q	1.81

Table S5. Frequencies of amino acids at variable positions among Rosetta CoupledMoves designs of P450pra optimized for *pro-R* compactin binding.

**Prava-epi1 (*pro-R*)[a]**										
**Position**	**1st**	**(%)**	**2nd**	**(%)**	**3rd**	**(%)**	**4th**	**(%)**	**5th**	**(%)**
F76	F	52.0	V	31.4	I	8.48	T	2.93	L	1.95
P80	A	34.1	G	26.9	S	15.6	H	5.54	C	4.90
T95	I	83.2	V	15.1	T	0.94	R	0.65	A	0.07
M179	M	46.1	A	25.4	L	12.9	G	3.89	C	3.84
V180	A	23.7	I	21.6	V	16.0	C	14.3	T	14.0
V182	T	26.7	A	24.2	C	12.2	V	9.97	S	9.51
T286	V	47.4	I	20.5	L	10.3	W	8.68	H	7.94
A388	G	45.7	A	38.7	S	8.01	T	5.99	P	0.56
**Prava-epi2 (*pro-R*)[a]**										
**Position**	**1st**	**(%)**	**2nd**	**(%)**	**3rd**	**(%)**	**4th**	**(%)**	**5th**	**(%)**
F76	F	26.4	V	24.8	H	9.61	T	7.71	I	5.05
P80	A	43.6	S	16.6	F	14.5	G	7.68	P	5.92
T95	I	55.1	V	44.1	T	0.58	A	0.13	R	0.00
M179	L	50.3	M	25.1	A	13.2	G	7.99	C	1.41
V180	A	28.5	V	28.1	T	26.9	C	8.80	S	5.77
V182	V	34.2	A	15.9	T	14.1	G	10.8	S	9.00
T286	L	70.6	I	16.8	F	6.44	M	2.49	V	2.03
A388	A	41.7	W	16.2	M	13.3	T	10.3	G	5.70

[a] Prava-epi1 and Prava-epi2 refer to starting structures obtained by manual repositioning of bound compactin, and a structure obtained by docking of compactin, respectively.

`

Table S6. Comparison of MD-predicted and experimental epimeric excess (e.e.) values. Percentages indicate the fraction of snapshots in which the enzyme-substrate complex passes the NAC criteria.

22ns × 5 seeds	prava NACs (%)	6-epi-prava NACs (%)	e.e. predicted (%)	e.e. experimental (%)
wild-type CYP105AS1	0.000	4.156	100 (epi)	78 (epi)
P450pra	0.087	0.000	100 (prava)	90 (prava)
P450pra100	0.172	0.000	100 (prava)	>99 (prava)
P450pra+V180M	0.020	0.000	100 (prava)	94 (prava)
P450pra +T95F	0.015	0.000	100 (prava)	90 (prava)

Table S7. NAC frequencies for hydrogens on different carbon atoms. Percentages indicate the fraction of snapshots in which the enzyme-substrate complex passes the NAC criteria. Figure S12 shows the positions of the relevant hydrogens in compactin.

22 ns × 5 seeds	H2 NACs (%)	H9 NAC (%)	H19 NAC (%)	H20 NACs (%)
wt-CYP105AS1	0.000	0.000	0.128	0.000
P450pra	0.033	0.031	0.000	3.808
P450pra100	0.051	0.003	0.000	0.372

Table S8. Dissociation energies of C-H bonds. Calculated C-H bond dissociation enthalpies (at 298 K) of compactin. The atoms are involved in C-H bonds that displayed near attack conformation along MD simulations. Figure S12 shows the positions of the relevant hydrogens in compactin.

Dissociation enthalpy	H2	H9	H19	H20	H98	H99
Total (kcal/mol)	96.5	80.7	100.1	99.7	74.4	74.4

Table S9. Comparison of predicted epimeric excess (e.e.) predicted by short MD simulations and experimental e.e. values. Numbers represent NAC percentages for production of pravastatin and 6-epi pravastatin as observed in 100 ps MD simulations.

100 ps × 50 seeds	prava NACs (%)	6-epi-prava NACs (%)	e.e. predicted (%)	e.e. experimental (%)
wt-CYP105AS1	0.000	17.386	100 (epi)	78 (epi)
P450pra	0.198	0.004	96 (prava)	90 (prava)
P450pra100	0.032	0.000	100 (prava)	>99 (prava)
P450pra+V180M	0.037	0.000	99 (prava)	94 (prava)
P450pra +T95F	0.114	0.002	99 (prava)	90 (prava)

`

Supplementary Figures

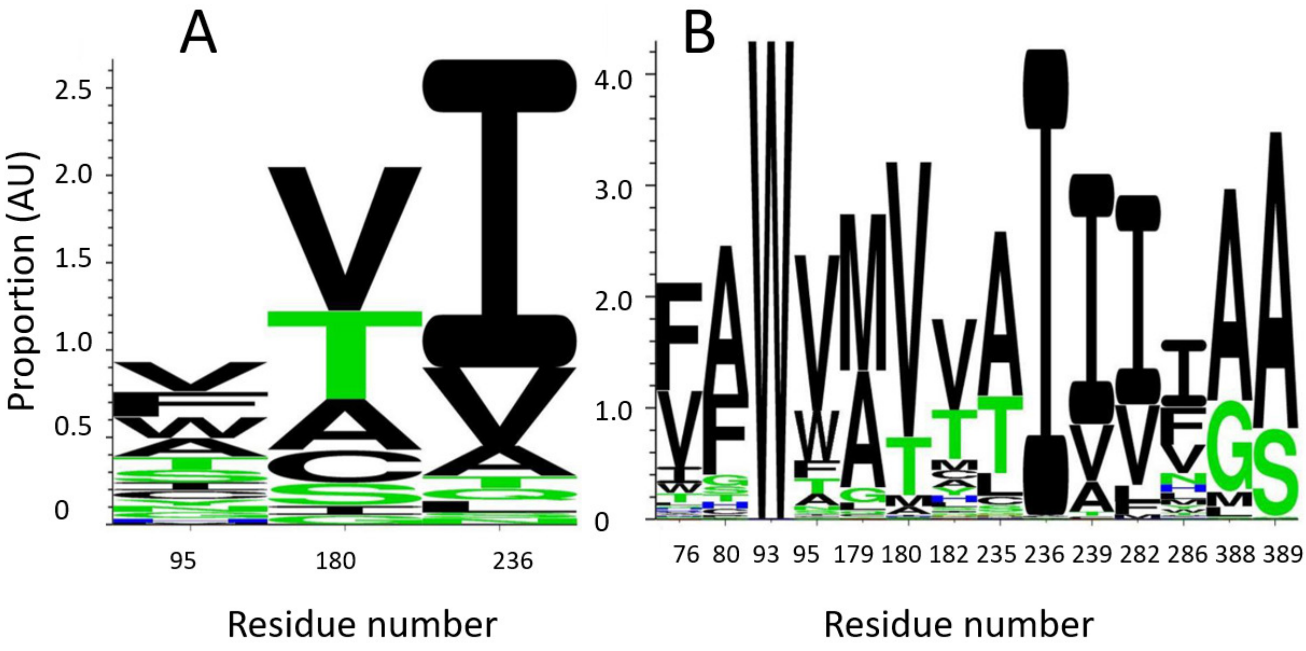

Figure S1. Frequency of occurrence of amino acids at specific positions in designs obtained by Rosetta CoupledMoves for binding of compactin in a pro-pravastatin mode. **A**, Variable positions 95, 180 and 236, with data obtained from top 251 low-energy designs. **B**, Eight variable positions as indicated, data obtained from top 200 low-energy designs. Picture constructed with WebLogo.[1]

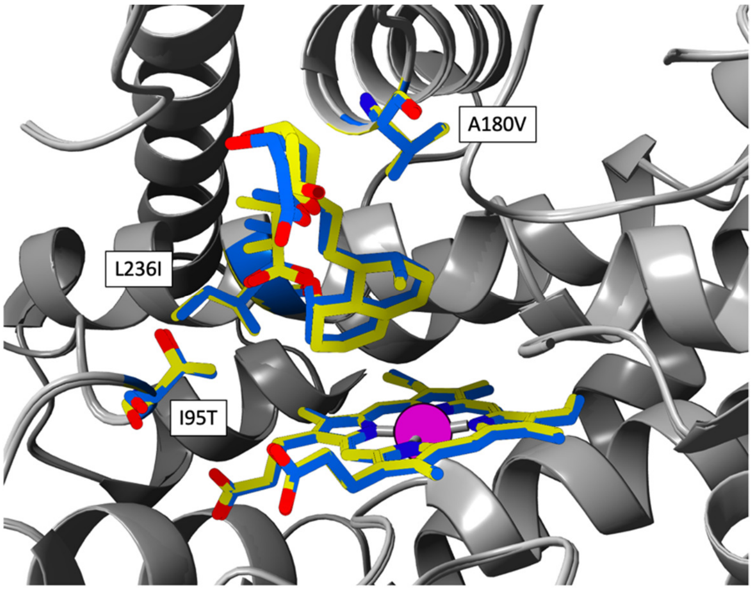

Figure S2. Alignment of the CoupledMoves-predicted structure and the P450pra crystal structure. The P450pra structure predicted by CoupledMoves is shown in blue and is aligned with the substrate-bound crystal structure in yellow (PDB ID: 4OQR). The RMSD of the two binding modes is 0.218 Å. The side chain of residues 95 and 180, shown in sticks, demonstrate the ability of CoupledMoves to correctly predict conformations of mutated side chains.

`

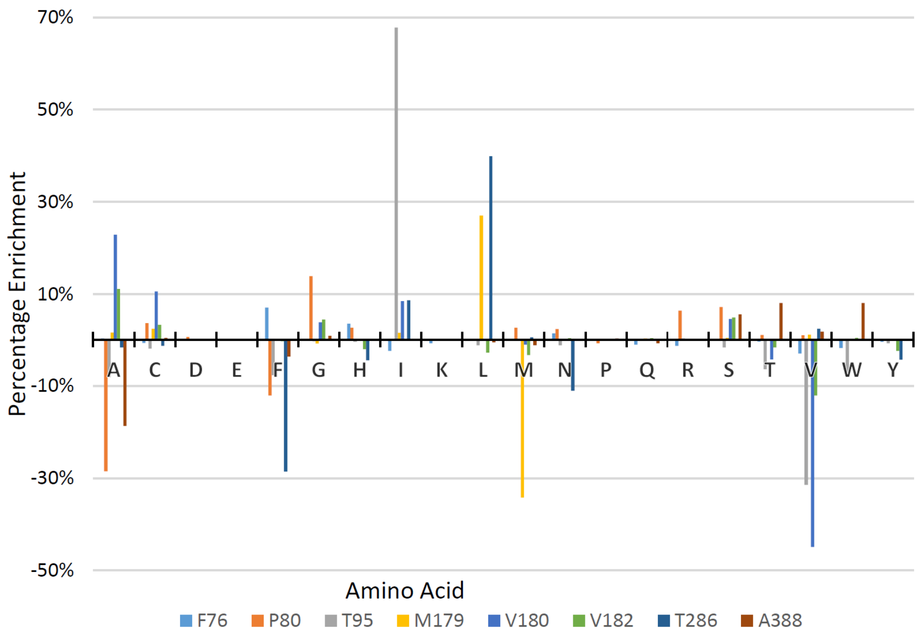

Figure S3. Differences in amino acid distribution at 8 positions among Rosetta CoupledMoves designs optimized for 6-epi-pravastatin (*pro-R* binding of compactin) and designs optimized for pravastatin (*pro-S* binding of compactin). Percentage enrichment is defined as the difference between the frequency of occurrence of a substitution in the set of *pro-R* and *pro-S* designs. Positive and negative Y-axis values indicate more frequent occurrence of amino acids among designs optimized for production of the 6-epi-pravastatin and pravastatin, respectively.

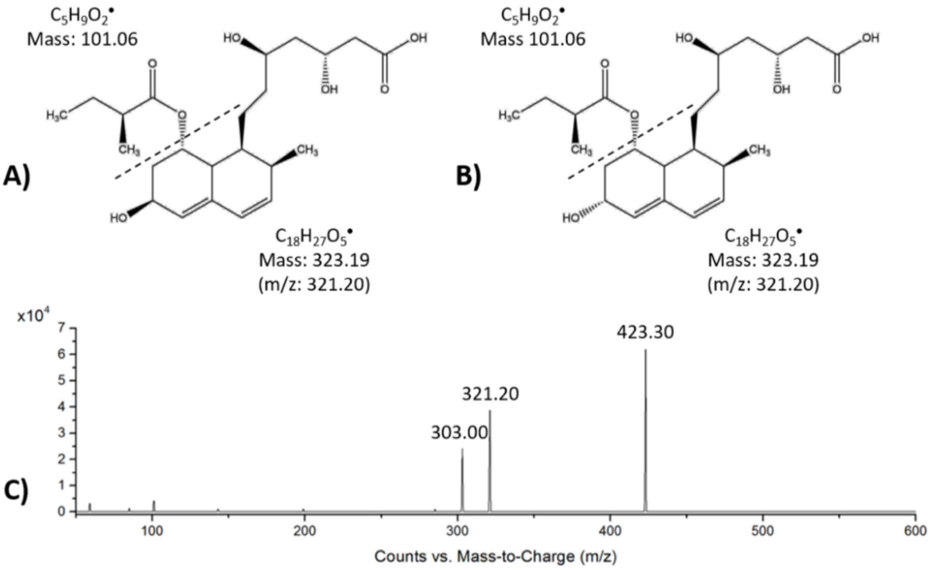

Figure S4. MS analysis of pravastatin and epi-pravastatin. A, pravastatin, m/z 424.24 (parent) or 323.19 (fragment). **B**, epi-pravastatin, giving the same fragment. **C**, MS spectrum of pravastatin.

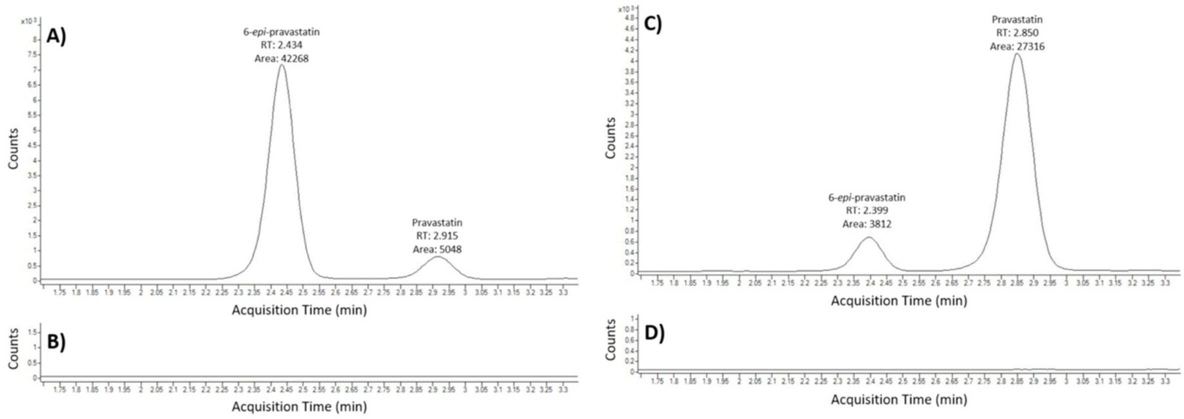

Figure S5. Epimeric composition as LC-MS QQQ data of pravastatin and epi-pravastatin mixtures produced by: **A)** wild-type CYPAS105; and **C)** P450pra. **B)** and **D)** show enzyme-free controls.

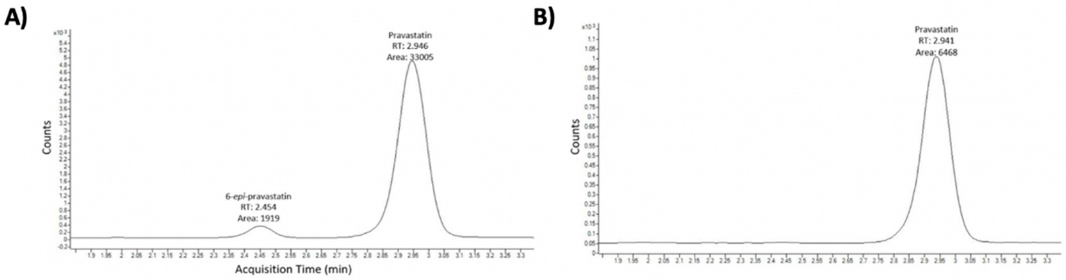

Figure S6. Epimeric composition of pravastatin and epi-pravastatin mixtures produced by P450 variants. **A)** P450pra +T95F; **B)** P450pra+T95F+V180M (P450pra100).

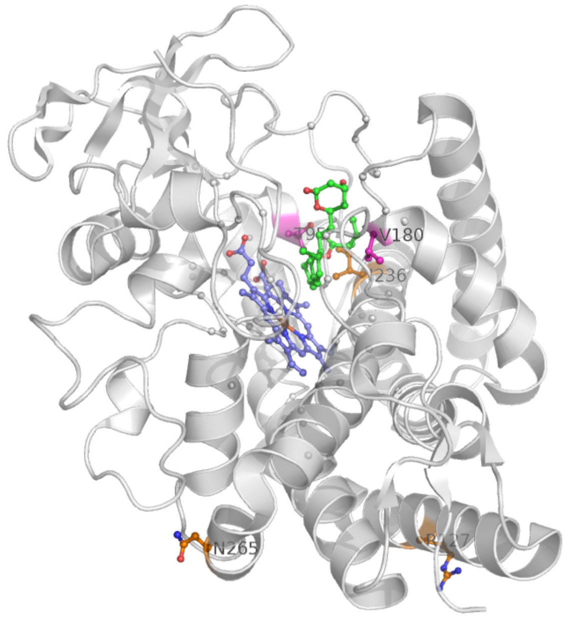

Figure S7. Location of mutations in P450pra100. Positions mutated from wild-type CYP105AS1 to P450pra100 are shown in orange and fuchsia. The mutations are T95F, Q127R, V180M, L236I, and A265N.

`

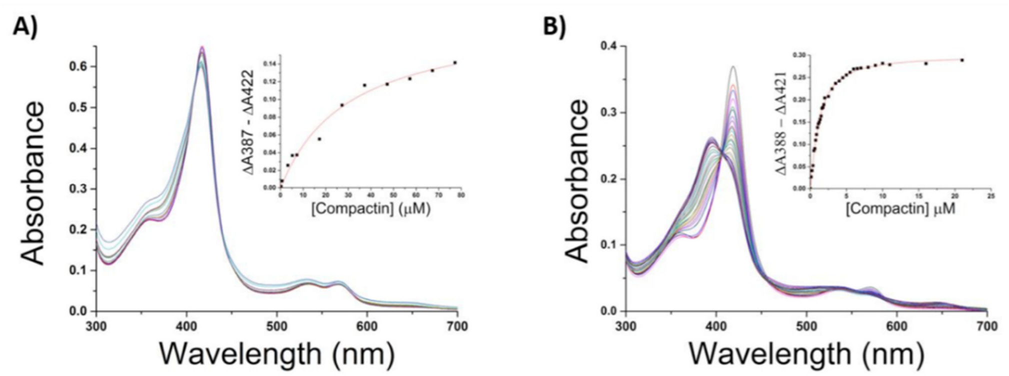

Figure S8. Compactin binding titrations with **A)** wild-type CYP105AS1, **B)** P450pra.

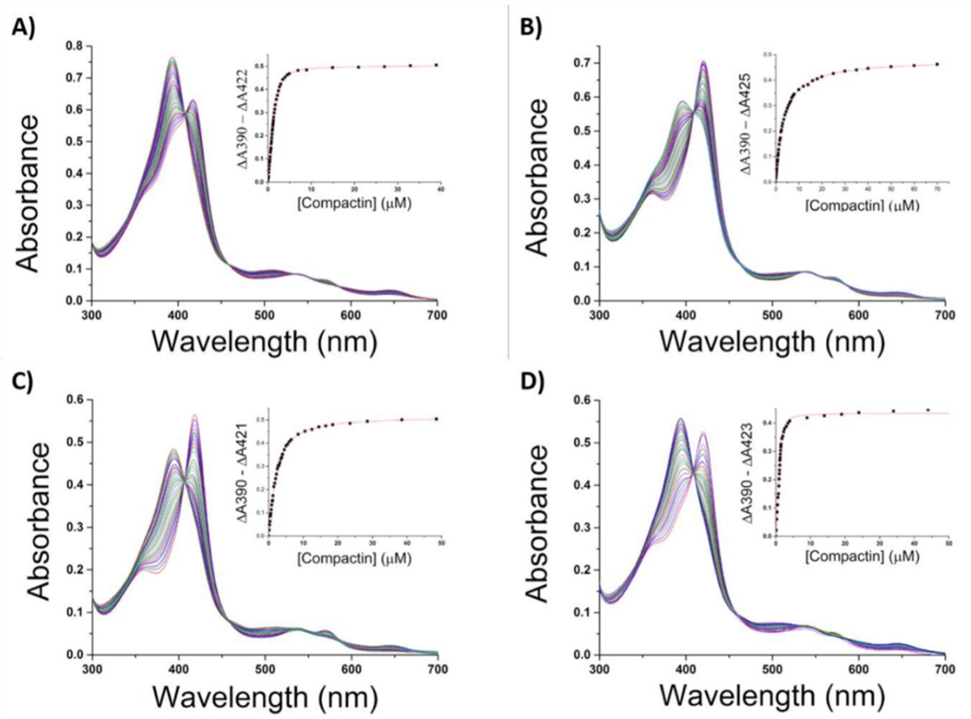

Figure S9. Compactin binding titrations with **A)** P450pra +T95F, **B)** P450pra +T95W, **C)** P450pra +V180M, **D)** P450pra +T95F+V180M (P450pra100).

`

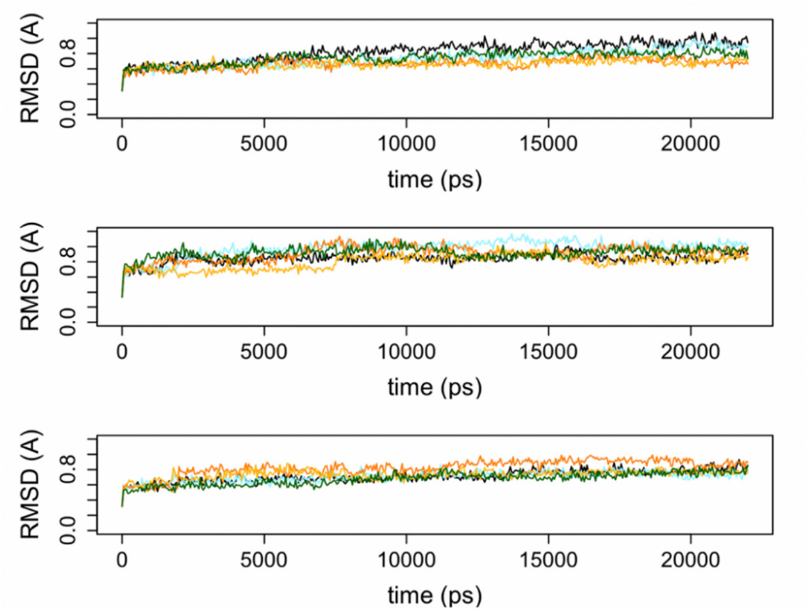

Figure S10. Stability of the enzyme-substrate complexes during MD simulations. The plots show the RMSD of the Cα atomic coordinates along 22 ns trajectories. For each enzyme-substrate complex, five independently seeded MD simulations are shown. **A)** wild-type CYP105AS1 with compactin bound; **B)** P450pra with compactin; **C)** P450pra100 with compactin.

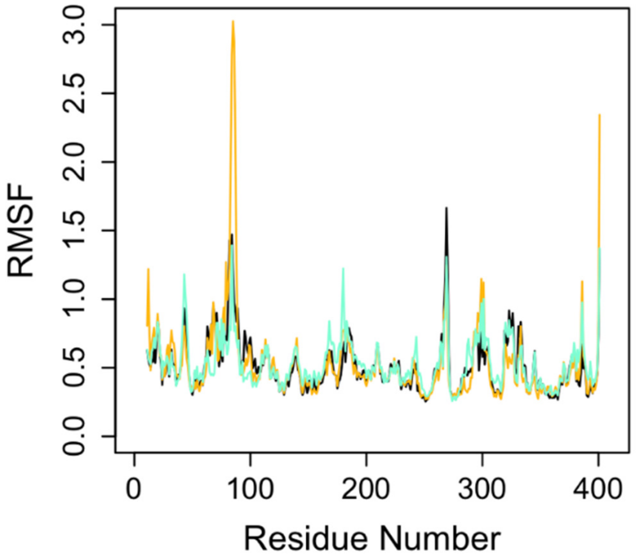

Figure S11. Root mean square fluctuation (RMSF) along MD simulations. The plots show RMSFs of enzyme-complexes along 22 ns MD simulations. Lines: wild-type CYP105AS1 with compactin bound (cyan), P450pra with compactin (yellow) and P450pra100 with compactin (black).

`

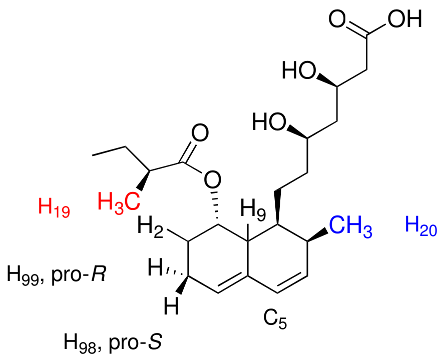

Figure S12. Compactin hydrogens adopting reactive conformations in MD simulations. Hydrogen positions H2, H9, H19, H20, H98, H99 were found to (transiently) adopt reactive conformations along MD simulations (Table S7). Table S8 shows the calculated dissociation energies of each of these C-H bonds. Pro-*S* and pro-*R* refer to the chirality of the product formed by replacement of the -H by -OH.

`

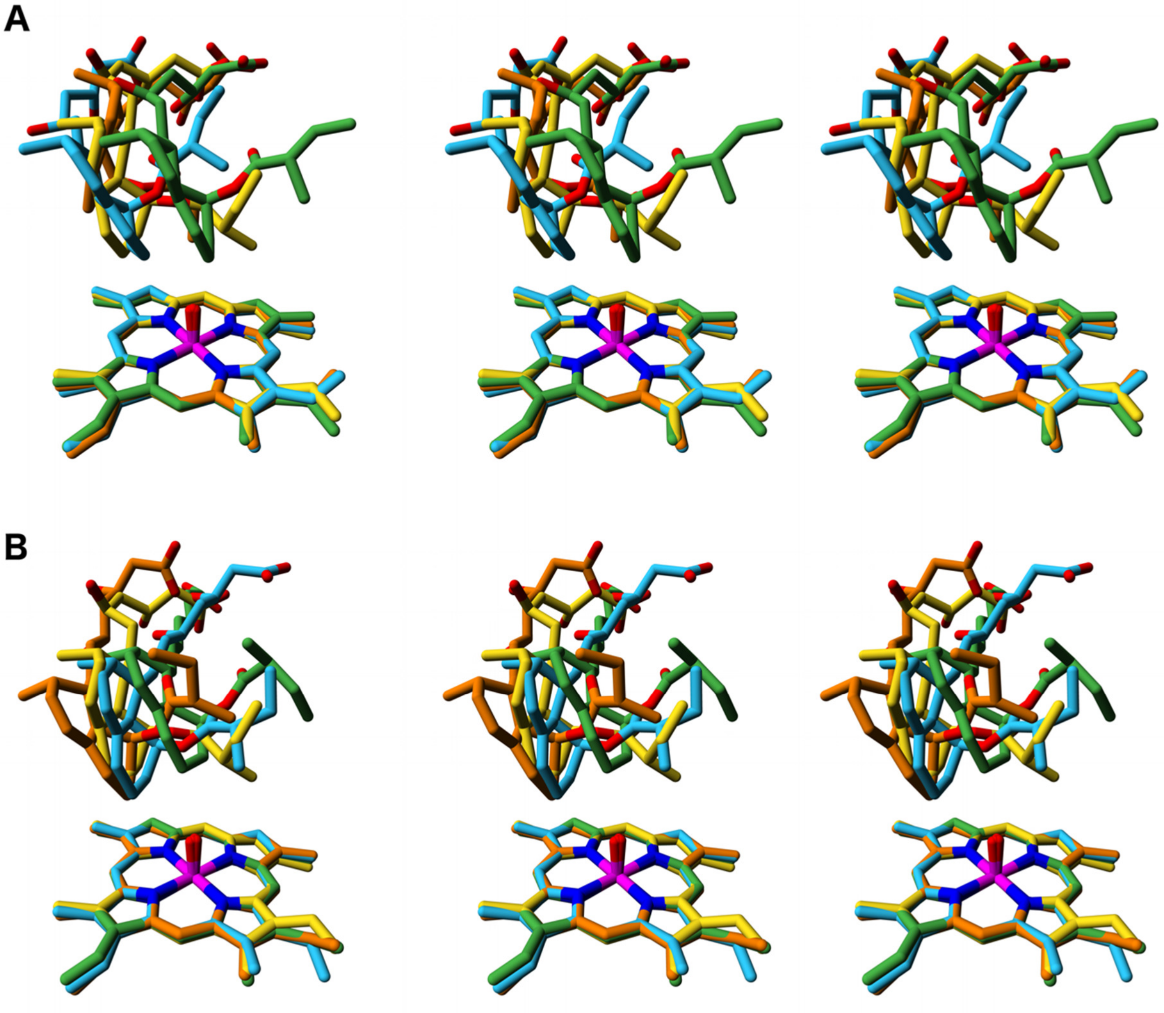

Figure S13. Diversity of sampled conformations in picosecond and nanosecond MD simulations. For MD simulations of the WT CYP105AS1, snapshots were collected during the production phase of 50 ×100 ps and 5 × 22 ns MD simulations, *i.e.* the last 50 ps and 20 ns, respectively. The propionate groups of the heme cofactors are omitted for clarity. To visualize the diversity of conformations, the three sampled conformations of the substrate that differ most from each other are shown. These conformations were obtained by superimposing all snapshots on the ring atoms of the heme group. Subsequently, the first selected snapshot was the one that differed most from the docked substrate orientation as evaluated by its RMSD over all substrate carbon atoms. The second snapshot was the one that differed most from the first snapshot while the third snapshot was the one that differed most form the first and second snapshot. **A)** conformations obtained during ps MD simulations compared to the docked orientation. The first, second, and third snapshots are shown in green, orange, and yellow carbon atoms while the docked orientation is shown with yellow carbon atoms. From the 550 snapshots obtained, 97 were most similar to the green snapshot, 247 were most similar to the orange snapshot, and 206 most similar to the cyan snapshot. The maximal RMSD between snapshots and their nearest displayed neighbor was 2.9 Å. **B)** Conformations obtained during nanosecond MD simulations. Of the 2005 snapshots, 642 were most similar to the snapshot with green carbon atoms, 786 were most similar to the one with orange, and 577 most similar to the snapshot labelled with cyan carbon atoms. The highest RMSD between displayed snapshots and other snapshots was 2.6 Å.

`

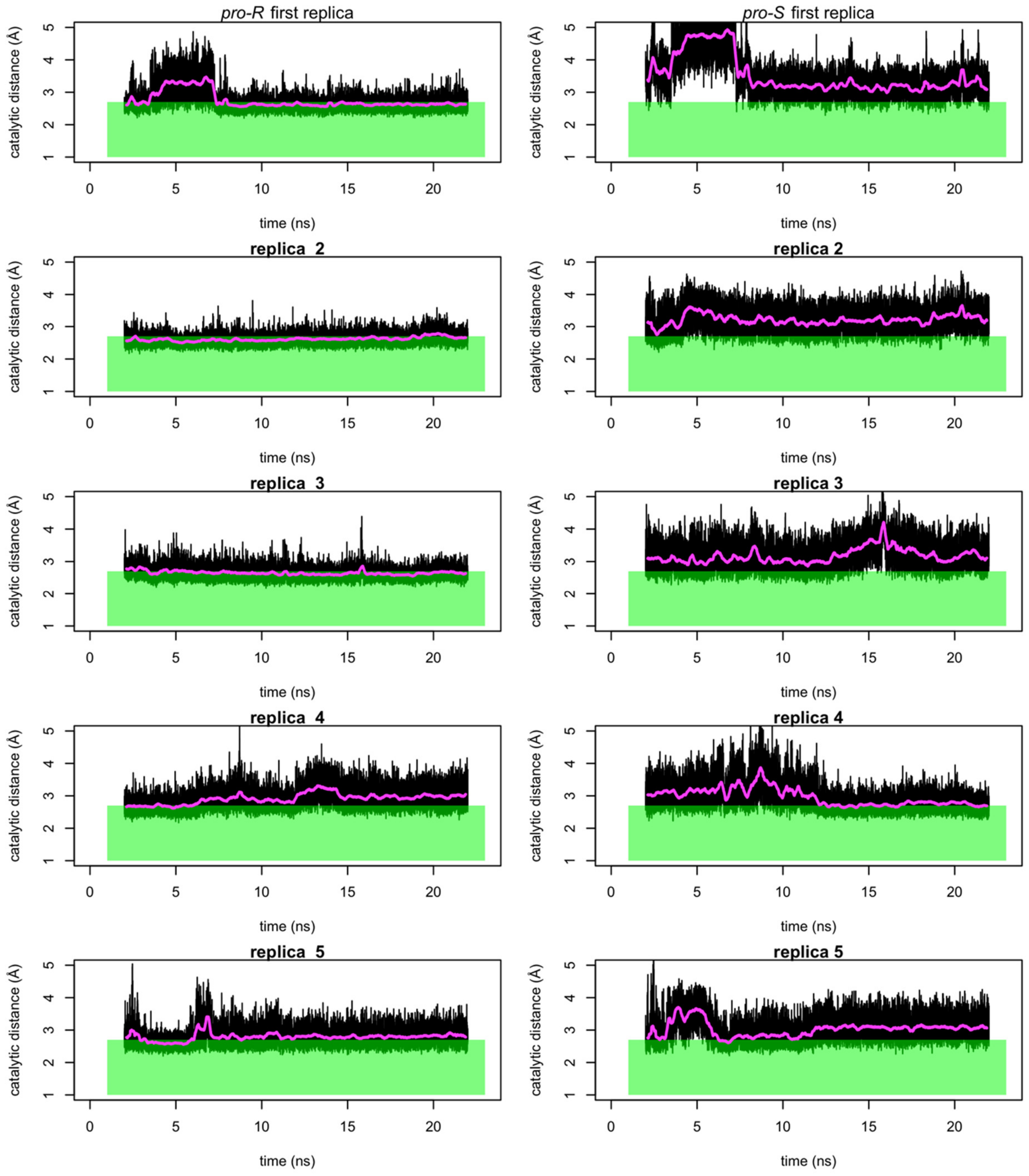

Figure S14. Sampling of the d_1 catalytic distance by nanosecond MD simulations. Shown is the distance between the oxygen of compound I and the *pro-R* hydrogen (left panels) and *pro-S* hydrogen (right panels) of compactin bound to P450wt. The time-series of points was gathered during the 2-22 ns production phase of the MD simulations with 1 ps intervals. Shown in magenta is a rolling mean with a spread of 250 ps. A green shade indicates where the catalytic distance is within NAC criteria (< 2.7 Å). In the starting structures of all MD simulations, the docked enzyme-substrate complex, the corresponding distances are 2.78 Å for the *pro-R* hydrogen and 4.10 Å for the *pro-S* hydrogen.

`

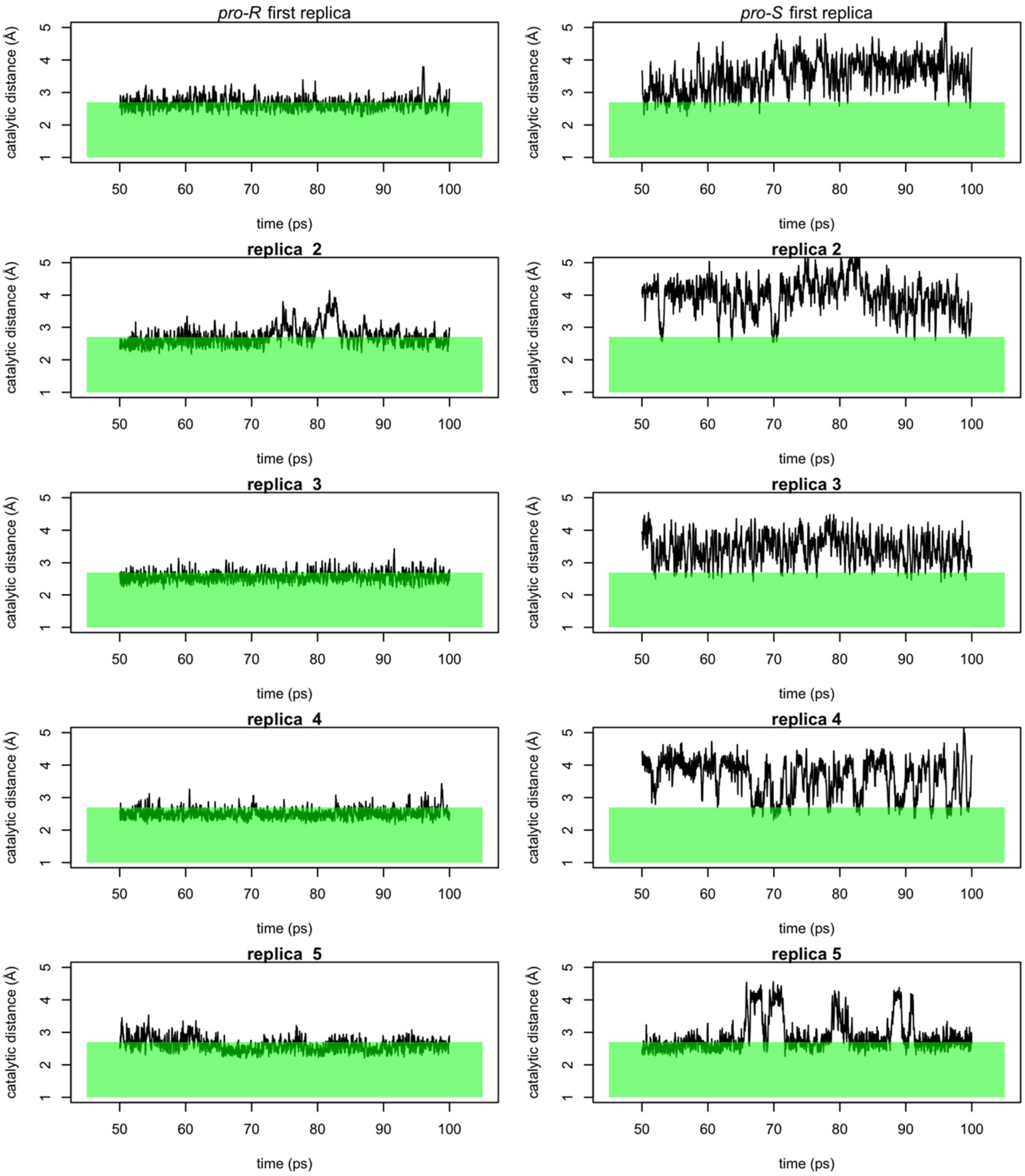

Figure S15. Sampling of the d_1 catalytic distances by picosecond MD simulations. Shown is the distance between the oxygen of compound I and the *pro-R* and *pro-S* hydrogens of compactin bound to P450wt. The time-series of points was gathered during the 50-100 ps production phase of the MD simulations with 20 fs intervals. A green shade indicates where the catalytic distance is within NAC criteria (< 2.7 Å). Only the first 5 of in total 50 replica MD simulations are shown. In the starting structures of all MD simulations, the docked enzyme-substrate complex, the corresponding distances are 2.78 Å for the *pro-R* hydrogen and 4.10 Å for the *pro-S* hydrogen.

`

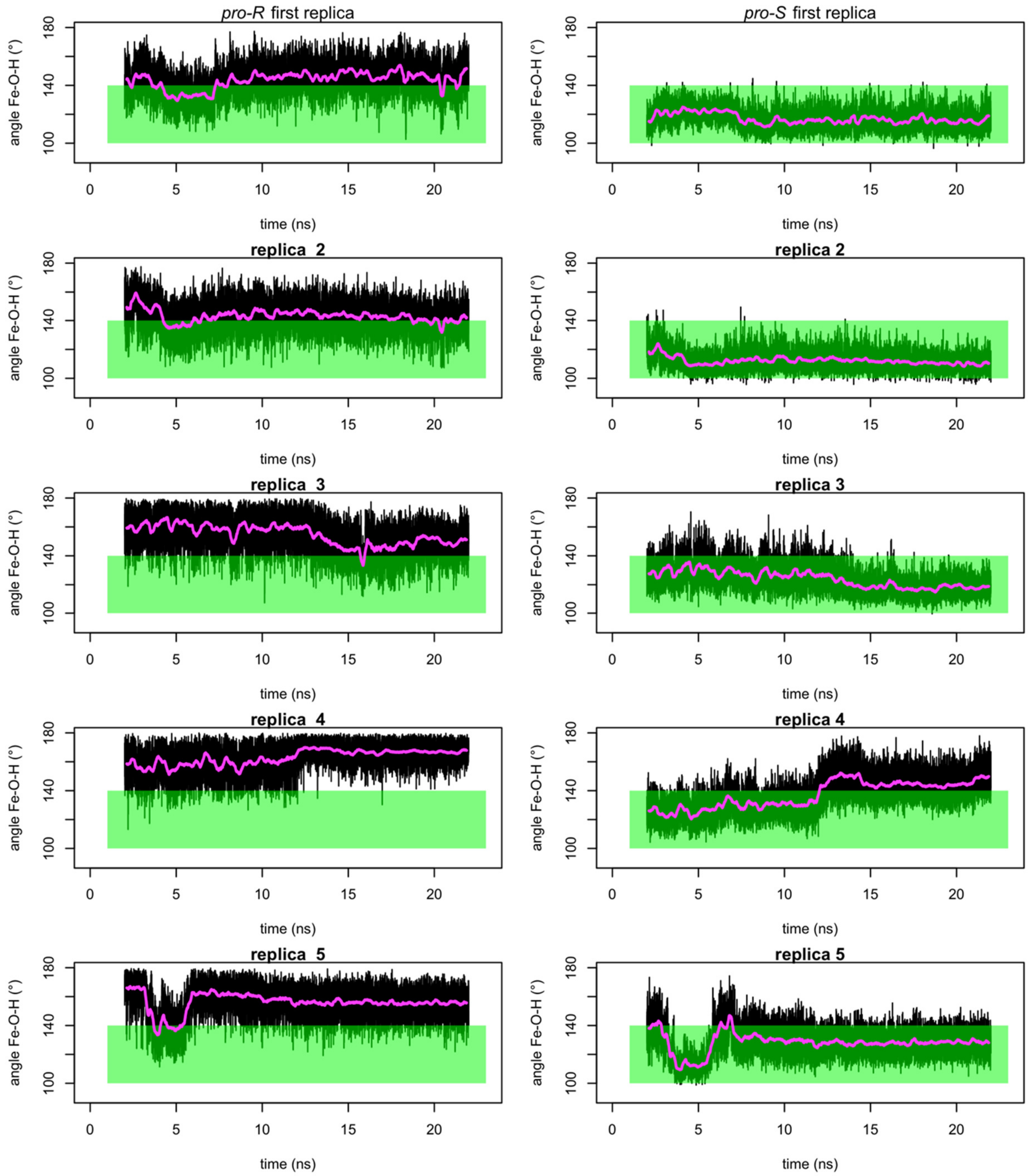

Figure S16. Sampling of the Θ_1 catalytic angle by nanosecond MD simulations. A green shade indicates where the angle is within NAC criteria (100-140°). See also legend to Figure S14. In the starting structures of all MD simulations, the docked enzyme-substrate complex, the corresponding angles are 126.5° for the *pro-R* hydrogen and 110.3° for the *pro-S* hydrogen.

`

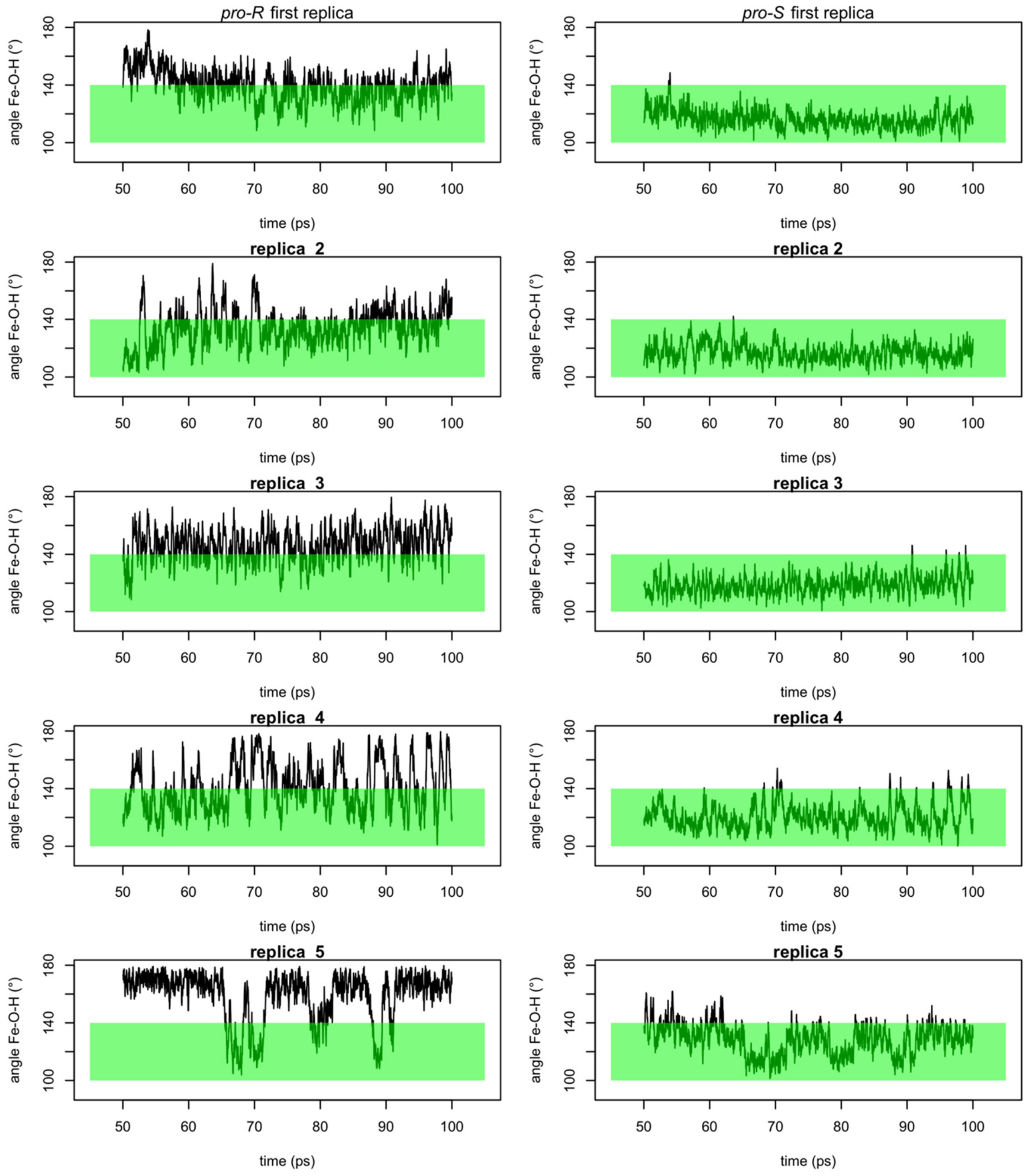

Figure S17. Sampling of the Θ_1 catalytic angle by picosecond MD simulations. A green shade indicates where the angle is within NAC criteria (100-140°). See also legend to Figure S15. In the starting structures of all MD simulations, the docked enzyme-substrate complex, the corresponding angles are 126.5° for the *pro-R* hydrogen and 110.3° for the *pro-S* hydrogen.

`

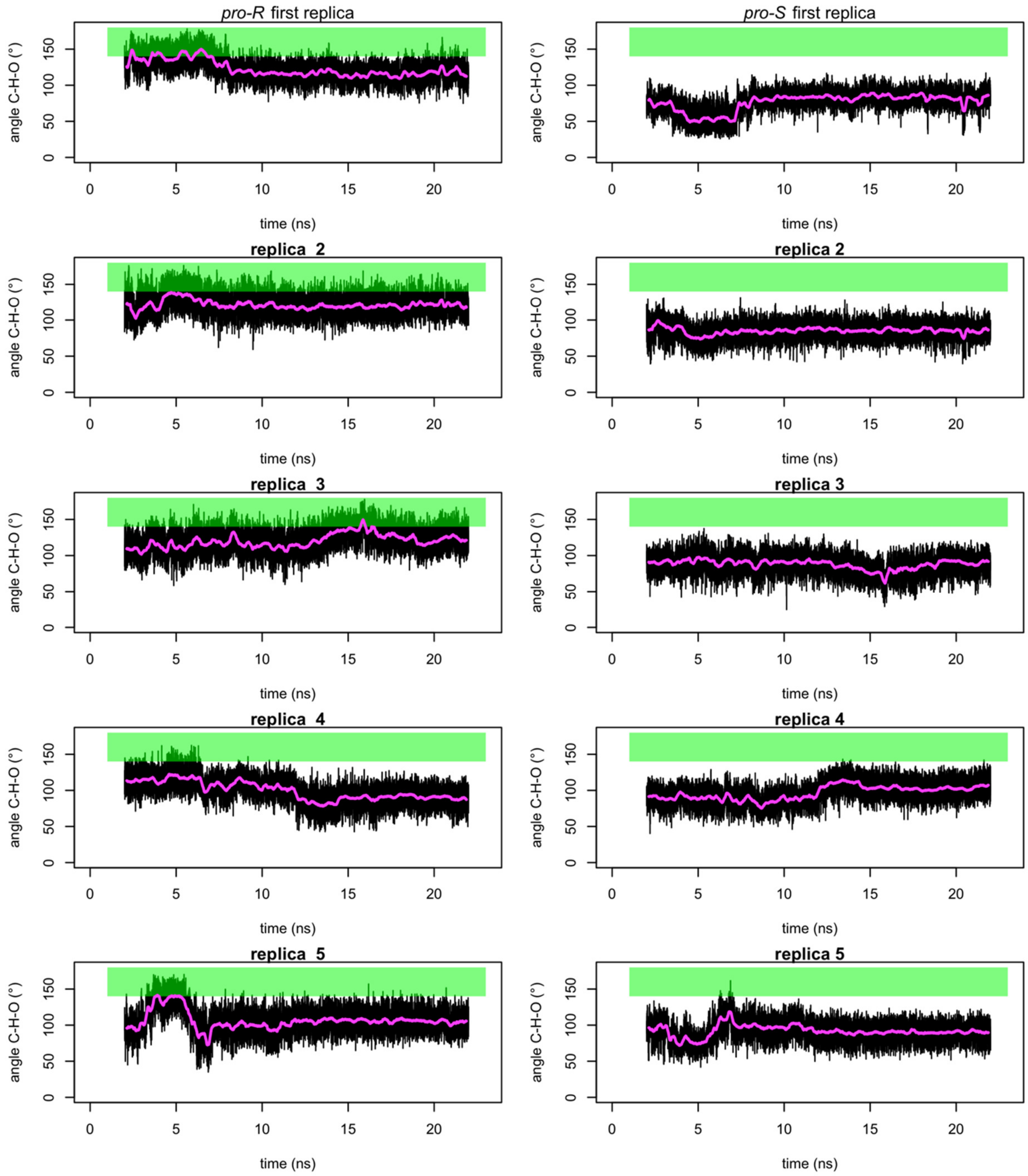

Figure S18. Sampling of the Θ_2 catalytic angle by nanosecond MD simulations. A green shade indicates where the angle is within NAC criteria (>140°). See also legend to Figure S14. In the starting structures of all MD simulations, the docked enzyme-substrate complex, the corresponding angles are 159.9° for the *pro-R* hydrogen and 61.8° for the *pro-S* hydrogen.

`

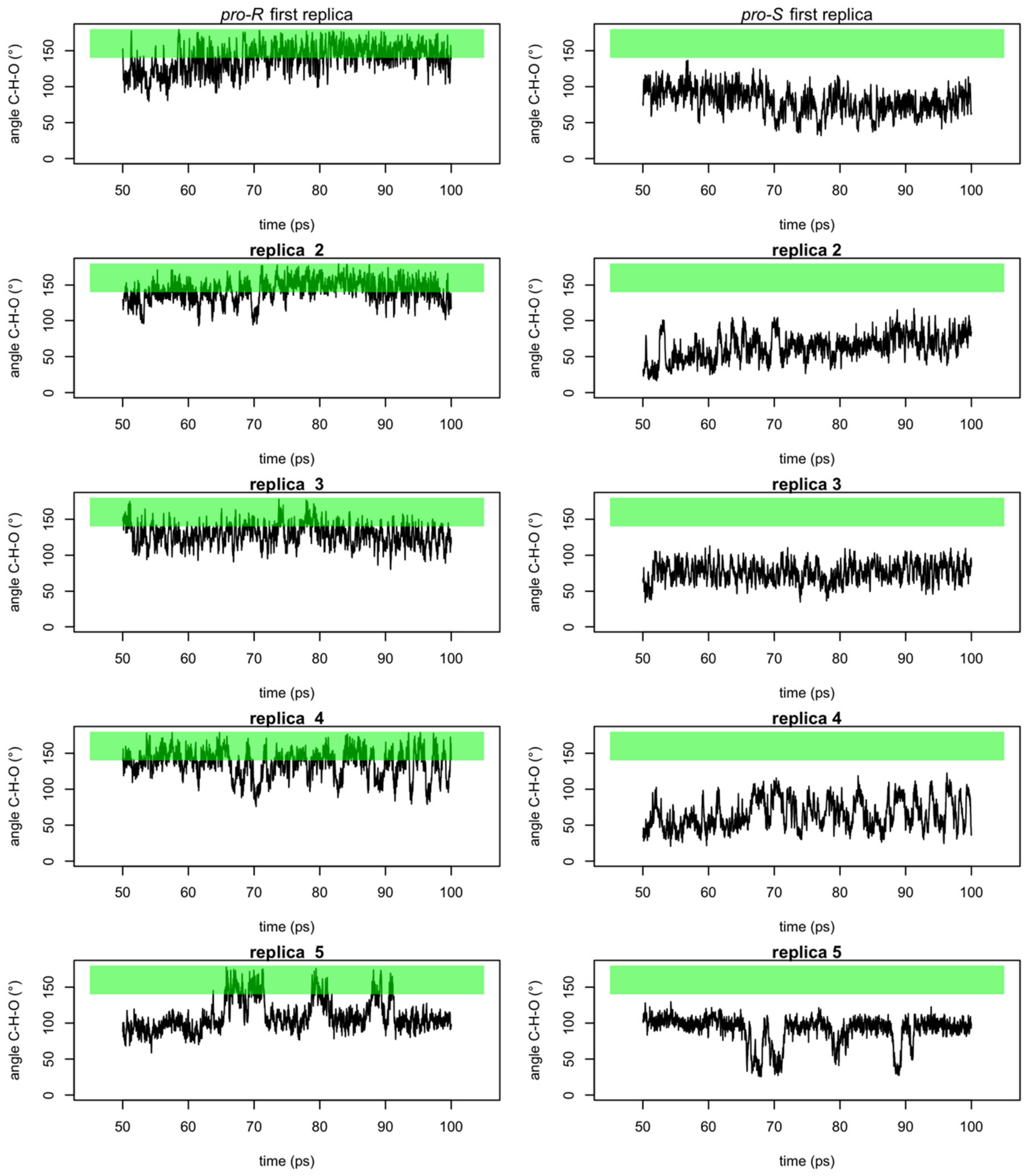

Figure S19. Sampling of the Θ$_2$ catalytic angle by picosecond MD simulations. A green shade indicates where the angle is within NAC criteria (>140°). See also legend to Figure S15. In the starting structures of all MD simulations, the docked enzyme-substrate complex, the corresponding angles are 159.9° for the *pro-R* hydrogen and 61.8° for the *pro-S* hydrogen.

References

(1) Crooks, G.E., Hon, G., Chandonia, J.M., Brenner, S.E. WebLogo: A Sequence Logo Generator. *Genome Research* **2004**, *14*, 1188-1190.
